# Supplementary material for: Development of optimized drug-like small molecule inhibitors of the SARS-CoV-2 3CL protease for treatment of COVID-19
Source: Nat Commun. 2022 Apr 7;13:1891. doi: 10.1038/s41467-022-29413-2 (PMC8989888; doi:10.1038/s41467-022-29413-2)
Supplement: Supplementary file 1 — Supplementary Information [file 41467_2022_29413_MOESM1_ESM.pdf]

Supplementary Information

Development of optimized drug-like small molecule inhibitors of the SARS-CoV-2 3CL protease for treatment of COVID-19

Hengrui Liu[1], Sho Iketani[2,3], Arie Zask[4], Nisha Khanizeman[1], Eva Bednarova[1], Farhad Forouhar[5], Brandon Fowler[1], Seo Jung Hong[6], Hiroshi Mohri[2], Manoj S. Nair[2], Yaoxing Huang[2], Nicholas E. S. Tay[1], Sumin Lee[1], Charles Karan[7], Samuel J. Resnick[6,8], Colette Quinn[9], Wenjing Li[9], Henry Shion[9], Xin Xia[4], Jacob D. Daniels[10], Michelle Bartolo-Cruz[4], Marcelo Farina[4,11], Presha Rajbhandari[4], Christopher Jurtschenko[9], Matthew A. Lauber[9], Thomas McDonald[9], Michael E. Stokes[4], Brett Hurst[12], Tomislav Rovis[1]*, Alejandro Chavez[6]*, David D. Ho[2]*, and Brent R. Stockwell[1,4]*

[1] Department of Chemistry, Columbia University, New York, NY, 10027, USA

[2] Aaron Diamond AIDS Research Center, Columbia University Irving Medical Center, New York, NY, 10032, USA

[3] Department of Microbiology and Immunology, Columbia University Irving Medical Center, New York, NY, 10032, USA

[4] Department of Biological Sciences, Columbia University, New York, NY, 10027, USA

[5] Herbert Irving Comprehensive Cancer Center, Columbia University Irving Medical Center, New York, NY, 10032, USA

[6] Department of Pathology and Cell Biology, Columbia University Irving Medical Center, New York, NY, 10032, USA

[7] Sulzberger Columbia Genome Center, Columbia University, New York, NY, 10032, USA

[8] Medical Scientist Training Program, Columbia University Irving Medical Center, New York, NY, 10032, USA

[9] Waters Corporation, 34 Maple Street, Milford, MA, 01757, USA

[10] Department of Pharmacology, Columbia University, New York, NY, USA.

[11] Department of Biochemistry, Federal University of Santa Catarina, Florianopolis, Santa Catarina, Brazil

[12] Institute for The Institute for Antiviral Research, Utah State University, Logan, UT, 84322, USA

***Corresponding Authors:** Tomislav Rovis (tr2504@columbia.edu), Alejandro Chavez (ac4304@cumc.columbia.edu), David D. Ho (dh2994@cumc.columbia.edu) and Brent R. Stockwell (bstockwell@columbia.edu)

Outline of Supplementary Information

Supplementary Figure 1-22 …………………………………………………………….…4

Supplementary Table 1-5……………………………………………………………….33

Supplementary Note 1…………………………………………………………….....…38

Reference of supplementary information……………………………………….....……54

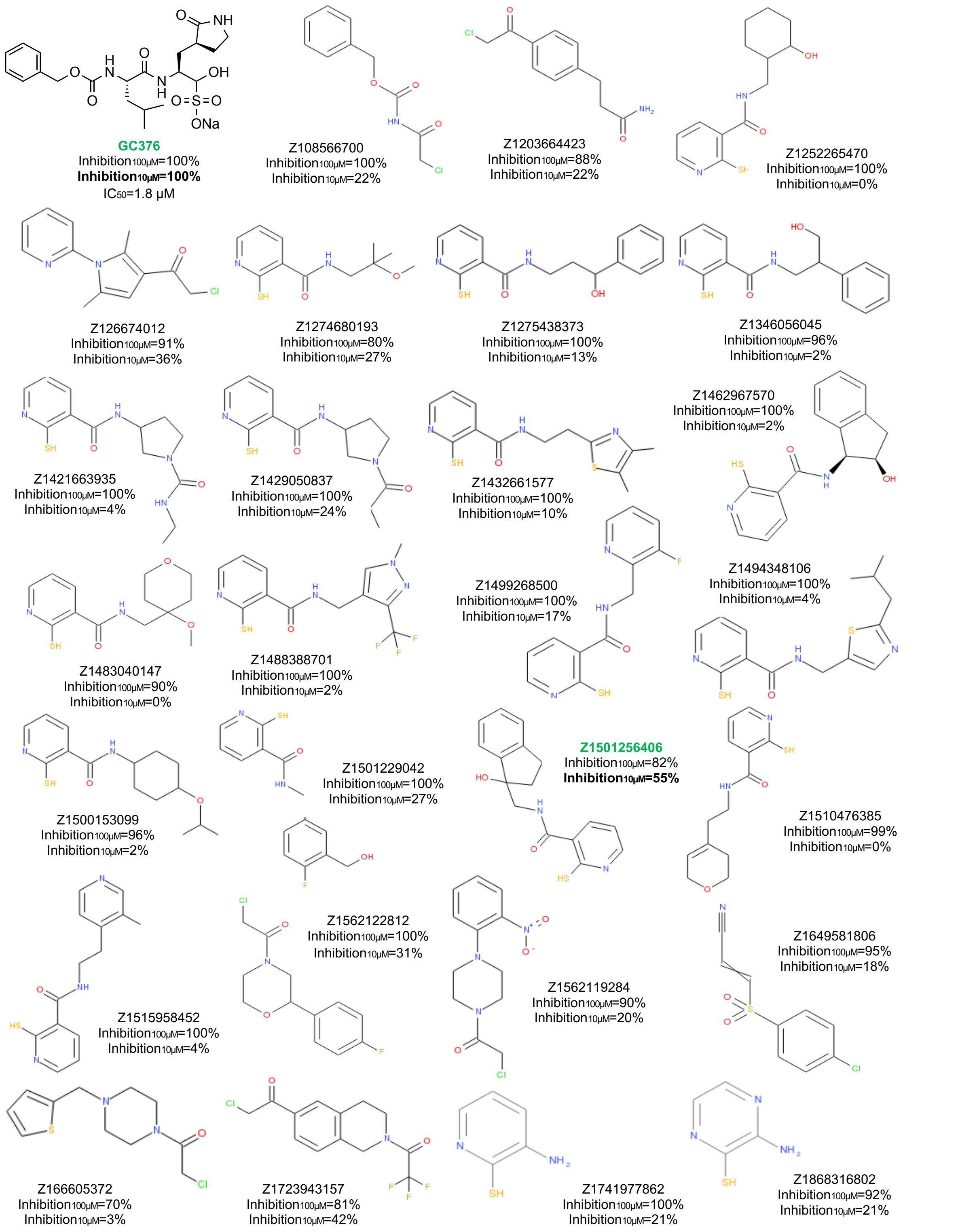

Supplementary Fig. 1. Evaluation of inhibitory activities of GC376 and the 27 hits from the screening of 7,247 electrophile fragments at indicated compound concentrations against SARS-CoV-2 3CL protease. Potent inhibitors (highlight in green) inhibit >50% protease activity at 10 μM (if IC50 < 10 μM). Detailed data with more concentrations and more information are reported in the source data file.

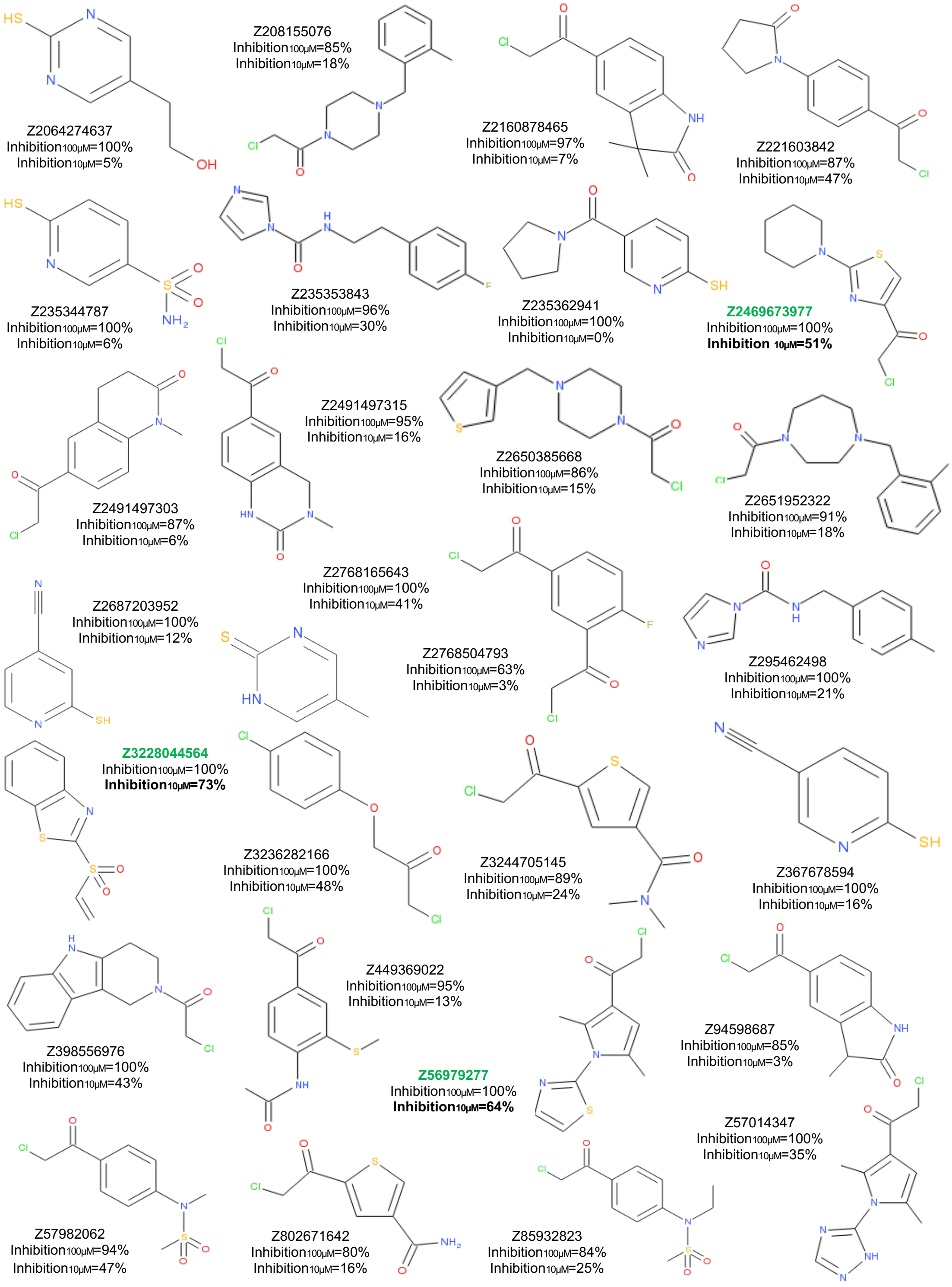

Supplementary Fig. 2. Evaluation of inhibitory activities of 28 hits from the screening of 7,247 electrophile fragments at indicated compound concentrations against SARS-CoV-2 3CL protease. Potent inhibitors (highlight in green) inhibit >50% protease activity at 10 μM (if IC50 < 10 μM). Detailed data with more concentrations and more information are reported in the source data file.

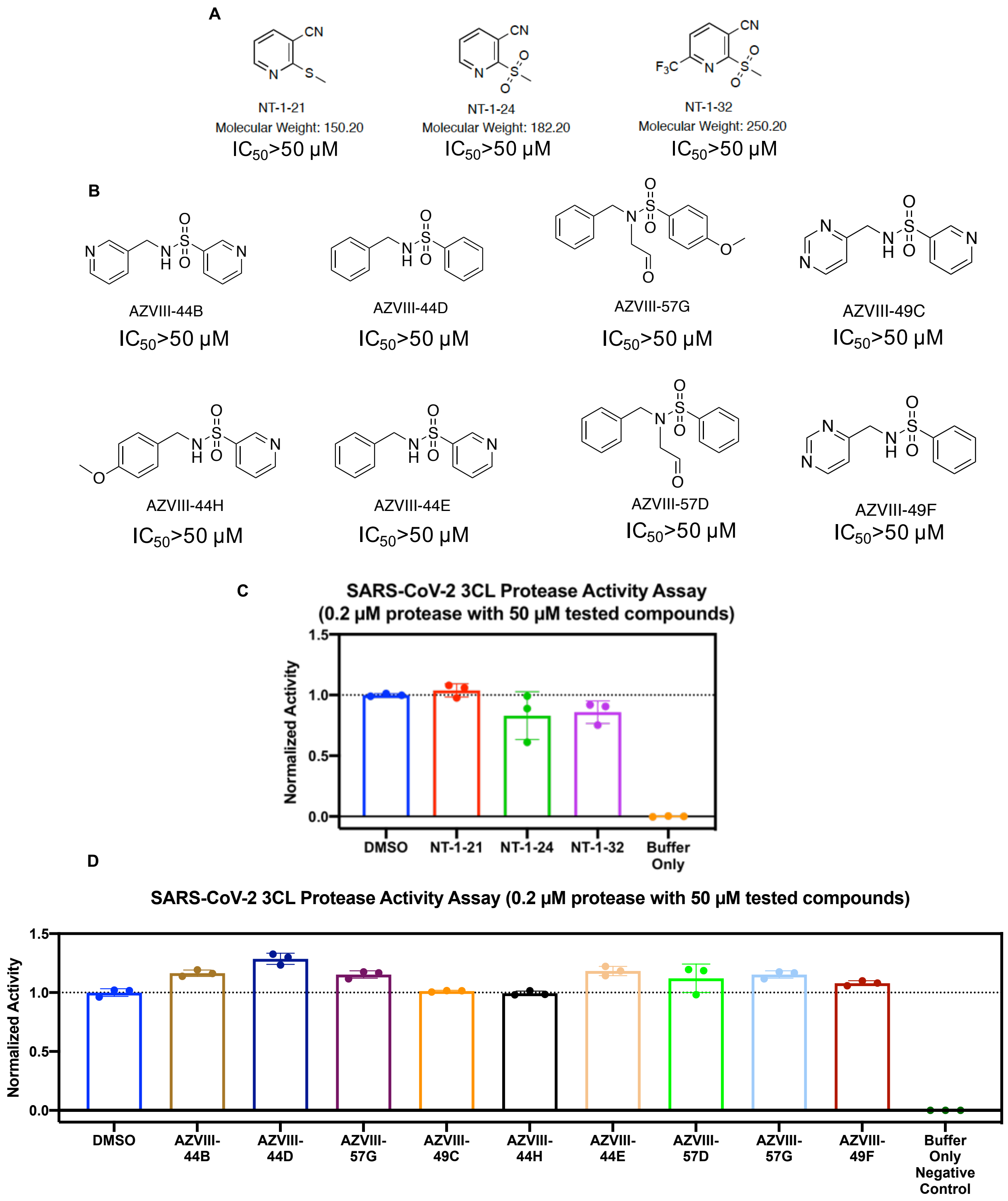

Supplementary Fig. 3. Evaluation of cysteine-reactive electrophiles as SRAS-CoV-2 3CL protease inhibitors. A, 2-sulfonylpyridines analogs. **B**, sulfonamide analogs. **C**, Test of 2-sufonylpyridines derivatives in the protease activity assay. Data are plotted as the mean ± s.d., n=3 biological replicates. **D**, Test of the sulfonamide derivatives in the

protease activity assay. Data are plotted as the mean ± s.d., n=3 biological replicates. Source data are provided as a Source Data file.

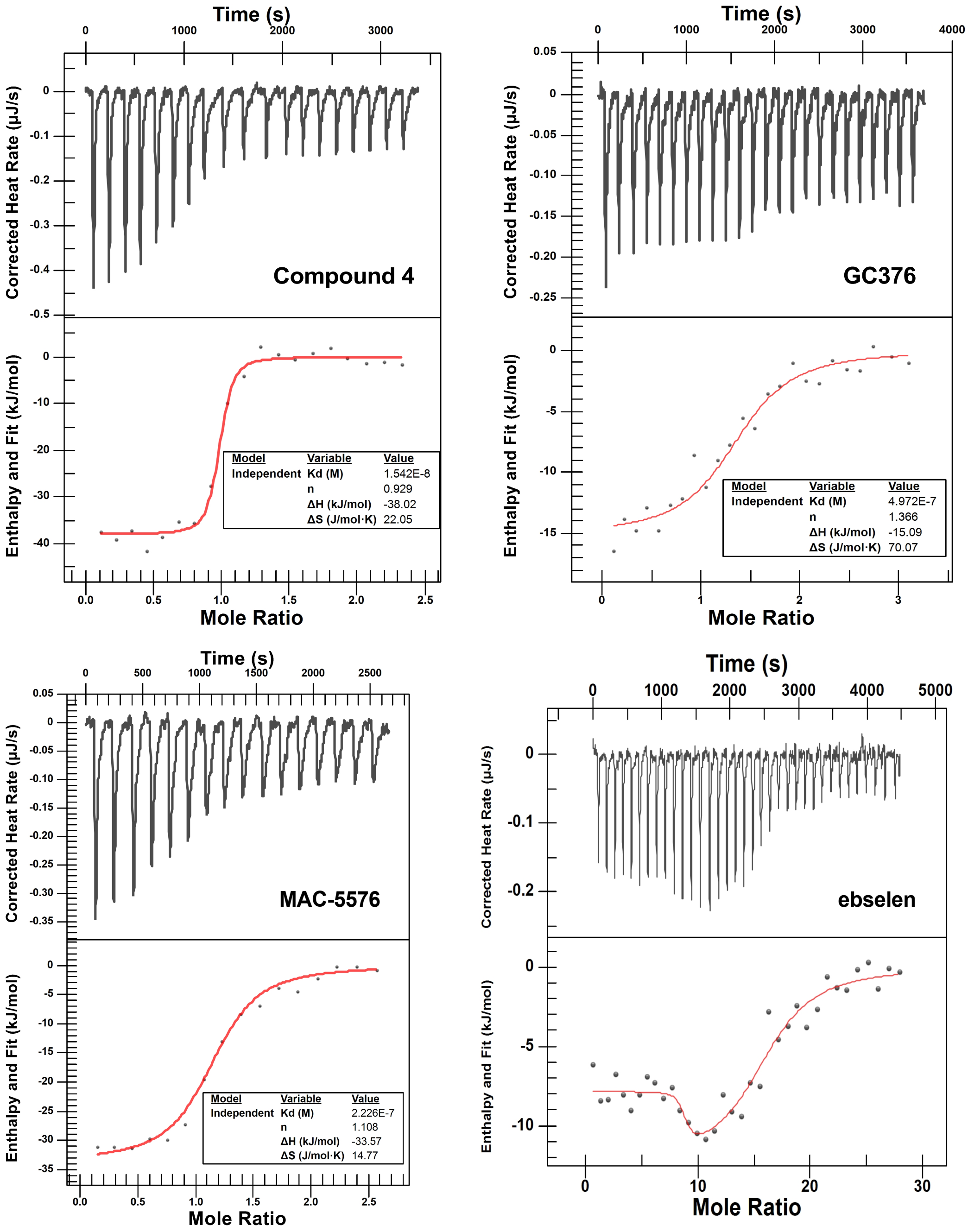

Supplementary Figure 4. Representative ITC analysis of the in vitro binding of compound 4, GC376, MAC-5576, and ebselen to SARS-CoV-2 3CL protease. Top: Raw titration data measured in µW. Each peak corresponds to a single 2.0 µL injection of 80 µM tested compound titrated into 6-8 µM 3CL-protease in 50 mM Tris Buffer, 1mM EDTA with 1% glycerol and 0.8% DSMO. Bottom: Integrated and normalized area under each peak plotted against mole ratio. Exothermic events are plotted as downward peaks here and for all subsequent figures. Each ligand was tested in biological duplicate. The average value along with standard error was reported in the text. The appropriate negative control of small molecule ligand into the running buffer was also performed.

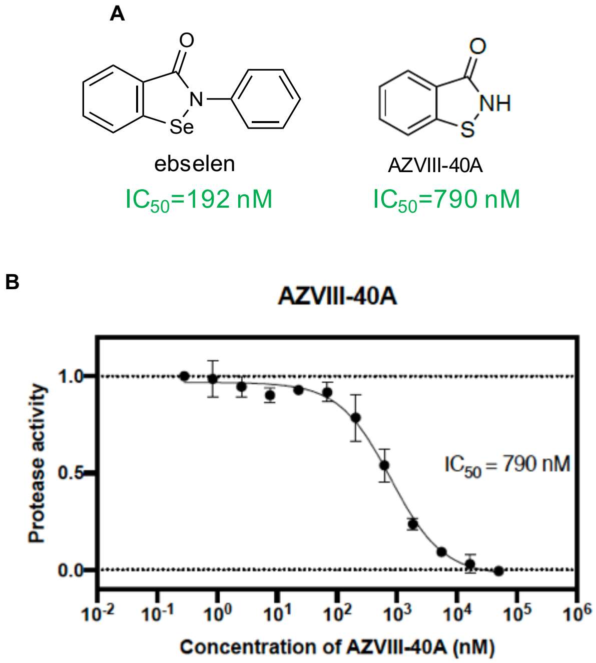

Supplementary Fig. 5. **Evaluation of an ebselen analog as SRAS-CoV-2 3CL protease inhibitor. A**, ebselen and its analog AZVIII-40A. **B**, Ebselen analog AZVIII-40A inhibited the activity of 200 nM SARS-CoV-2 protease with an IC50 value of 790nM. Data are plotted as the mean ± s.d., n=3 biological replicates. Source data are provided as a Source Data file.

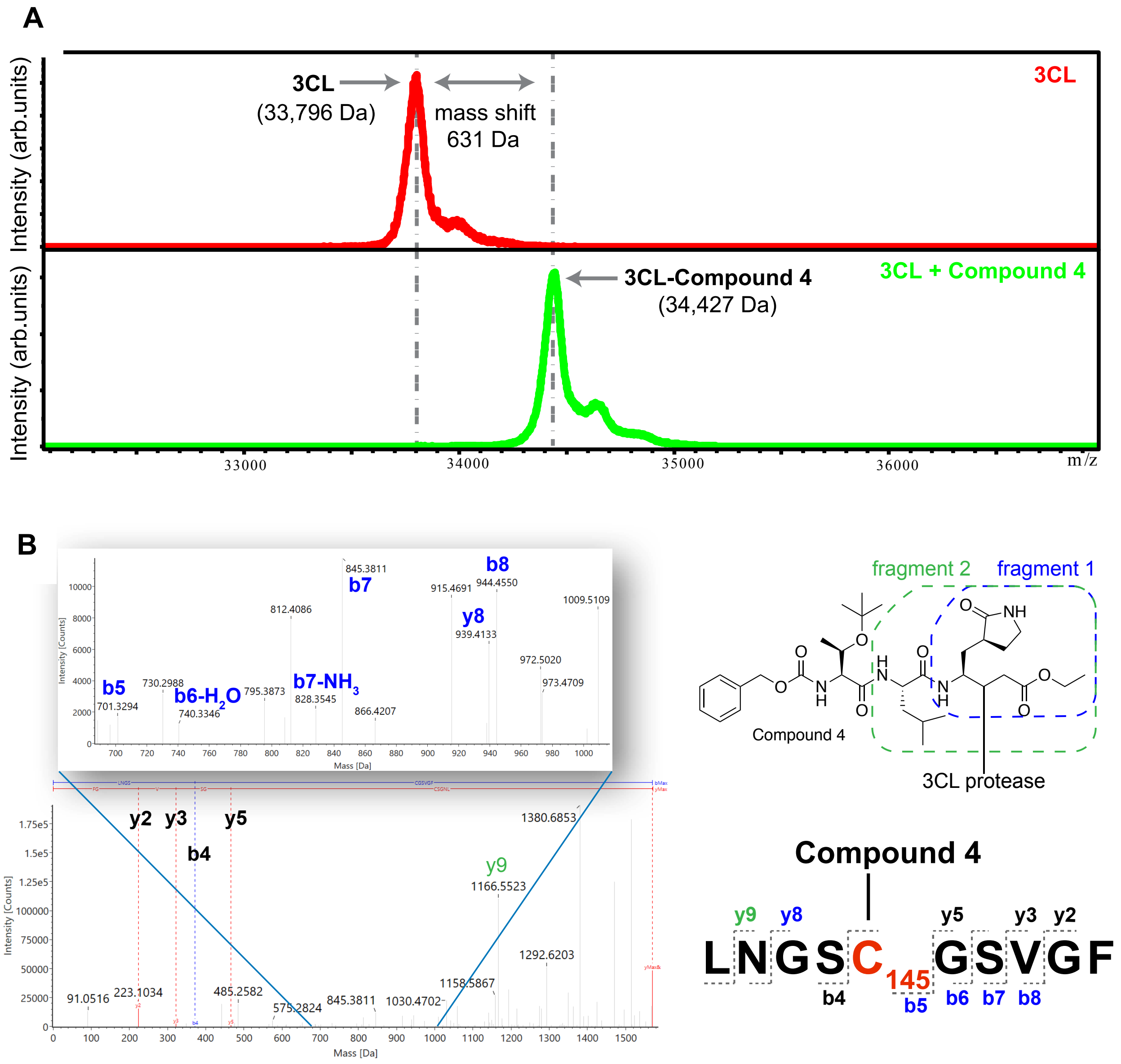

Supplementary Figure 6. Evaluation of Compound 4 for binding to SARS-CoV-2 3CL protease.

A, Intact protein MALDI MS of SARS-CoV-2 3CL protease (3CL, red) only and 3CL pre-incubated with compound 4 (green). **B**, LC–MS spectra of the compound 4 modification of SARS-CoV-2 3CL protease at residue C145. In the LC-MS analysis on digested 3CL-compound 4 complex, the peptide containing Cysteine 145 (LNGSCGSVGF) was analyzed for mass shift induced by binding of compound 4 fragment 1 (blue) and fragment 2 (green).

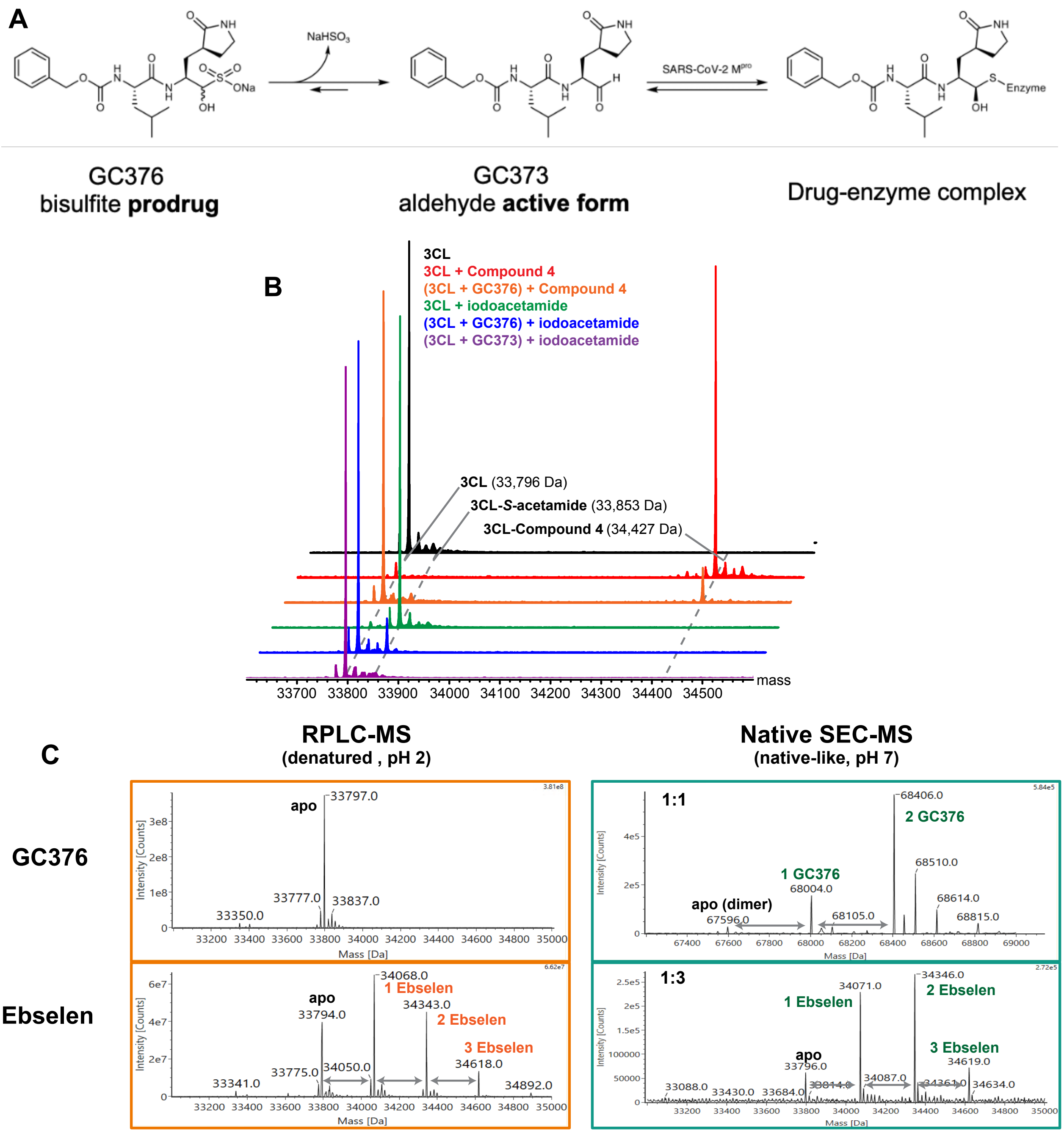

Supplementary Figure 7. Evaluation of GC376 and ebselen for binding to SARS-CoV-2 3CL protease.

A, Binding mechanism of GC376 to SARS-CoV-2 3CL. **B**, Reversed phase LC-ESI-MS (RPLC-MS) spectra of 3CL only (black), 3CL treated with 10 equivalent (eq) compound 4 for 1 hour at 4˚C (red), 3CL preincuated with 1 eq GC376 for 1 hour at 4˚C before treatment of 10 eq compound 4 for 1 hour at 4˚C (orange), 3CL treated with 10 eq iodoacetamide for 1 hour at 4˚C (green), 3CL preincuated with GC376 for 1 hour at 4˚C before treatment of 10 eq iodoacetamide for 1 hour at 4˚C (blue), and 3CL preincuated with 1 eq GC373 for 1 hour at 4˚C before treatment of 10 eq iodoacetamide for 1 hour at 4˚C (purple). **C**, RPLC-MS (pH=2) and Native size exclusion chromatography-ESI-MS (SEC-MS, pH ~7) spectra of 3CL-GC376 and 3CL-ebselen complex.

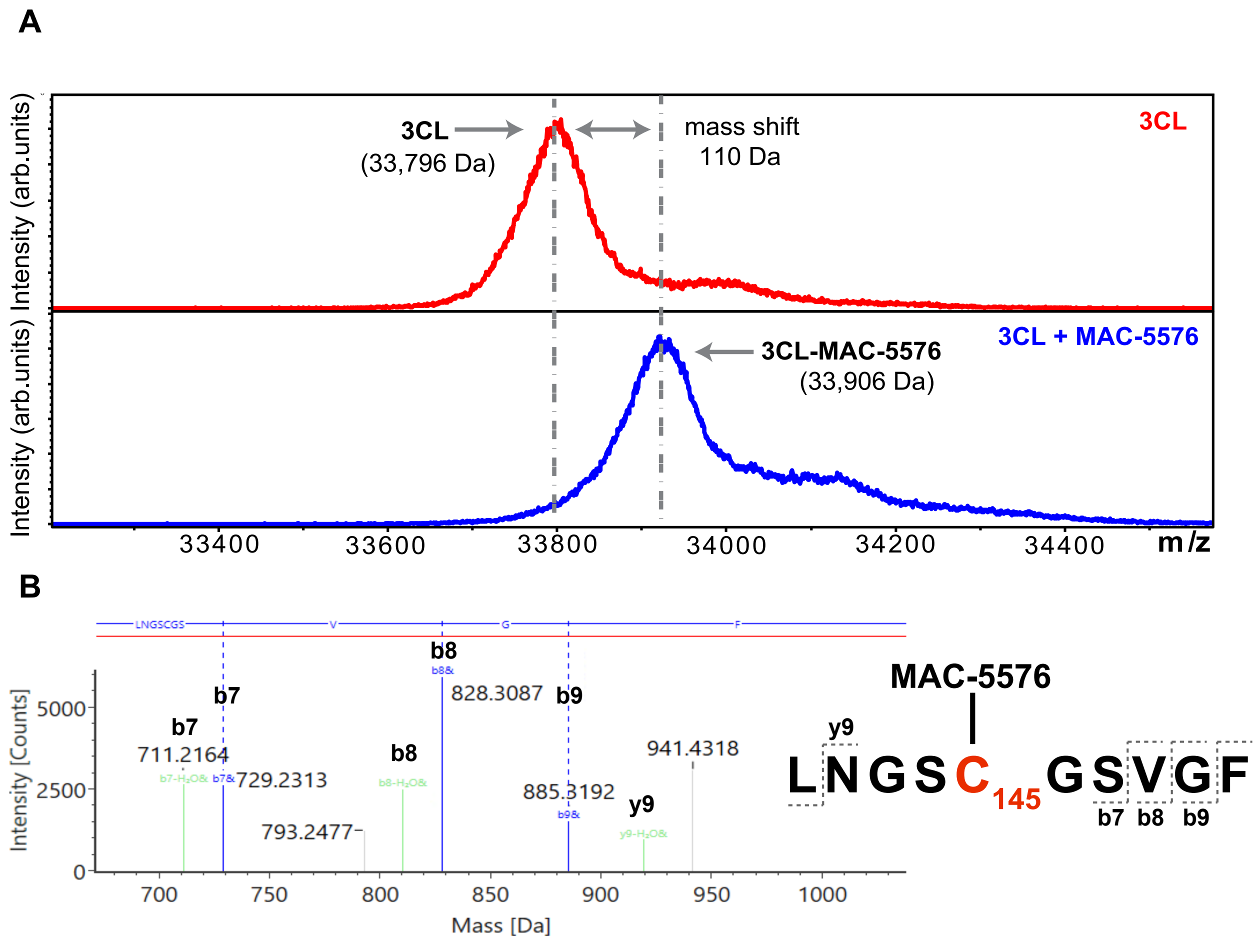

Supplementary Figure 8. Evaluation of MAC-5576 for binding to SARS-CoV-2 3CL protease.

A, Intact protein MALDI MS of SARS-CoV-2 3CL protease (3CL, red) only and 3CL pre-incubated with MAC-5576 (blue). **B**, LC–MS spectra of the MAC-5576 modification of SARS-CoV-2 3CL protease at residue C145. In the LC-MS analysis on digested 3CL-compound 4 complex, the peptide containing Cysteine 145 (LNGSCGSVGF) was analyzed for mass shift induced by binding of MAC-5576.

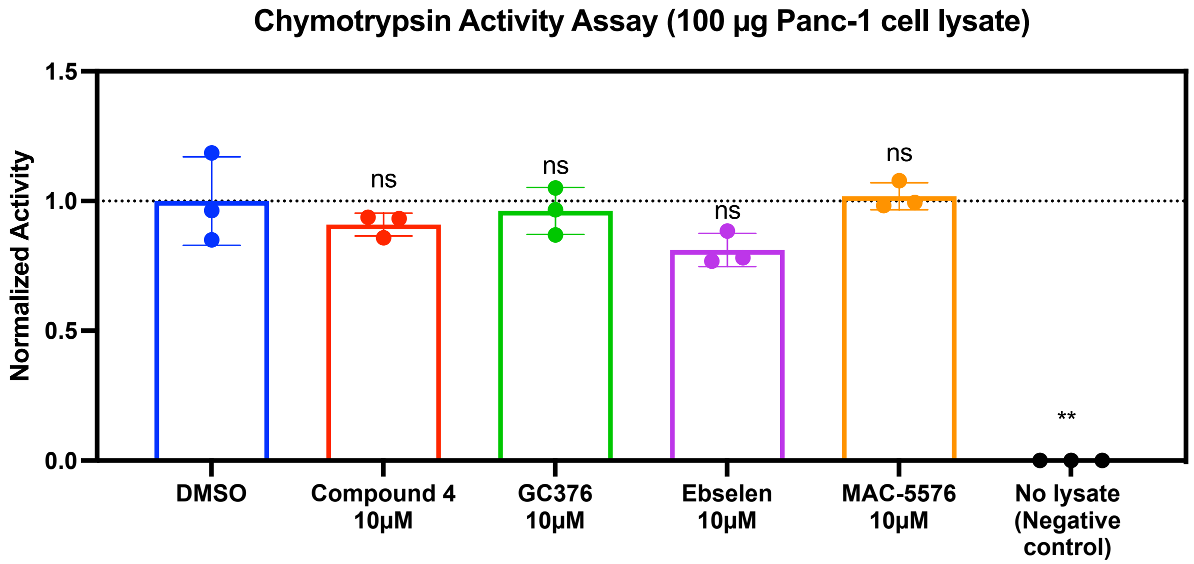

Supplementary Fig. 9. Selectivity of compound 4, GC376, MAC-5576, and ebselen for SARS-CoV-2 3CL proteases over human proteases. The in vitro biochemical activities of human chymotrypsin in the Panc-1 cell lysates treated with DMSO or compound 4, GC376, ebselen, and MAC-5576 were measured. Experiments were performed in triplicate. Data are plotted as the mean ± s.d., n = 3 biological replicates. Two-tailed t test was performed versus DMSO. P^{ns} of Compound 4 = 0.54, P^{ns} of GC376 = 0.82, P^{ns} of ebselen = 0.28, P^{ns} of MAC-5576 = 0.90, and P^{**} of no lysate = 0.0096, all versus DMSO control. Source data are provided as a Source Data file.

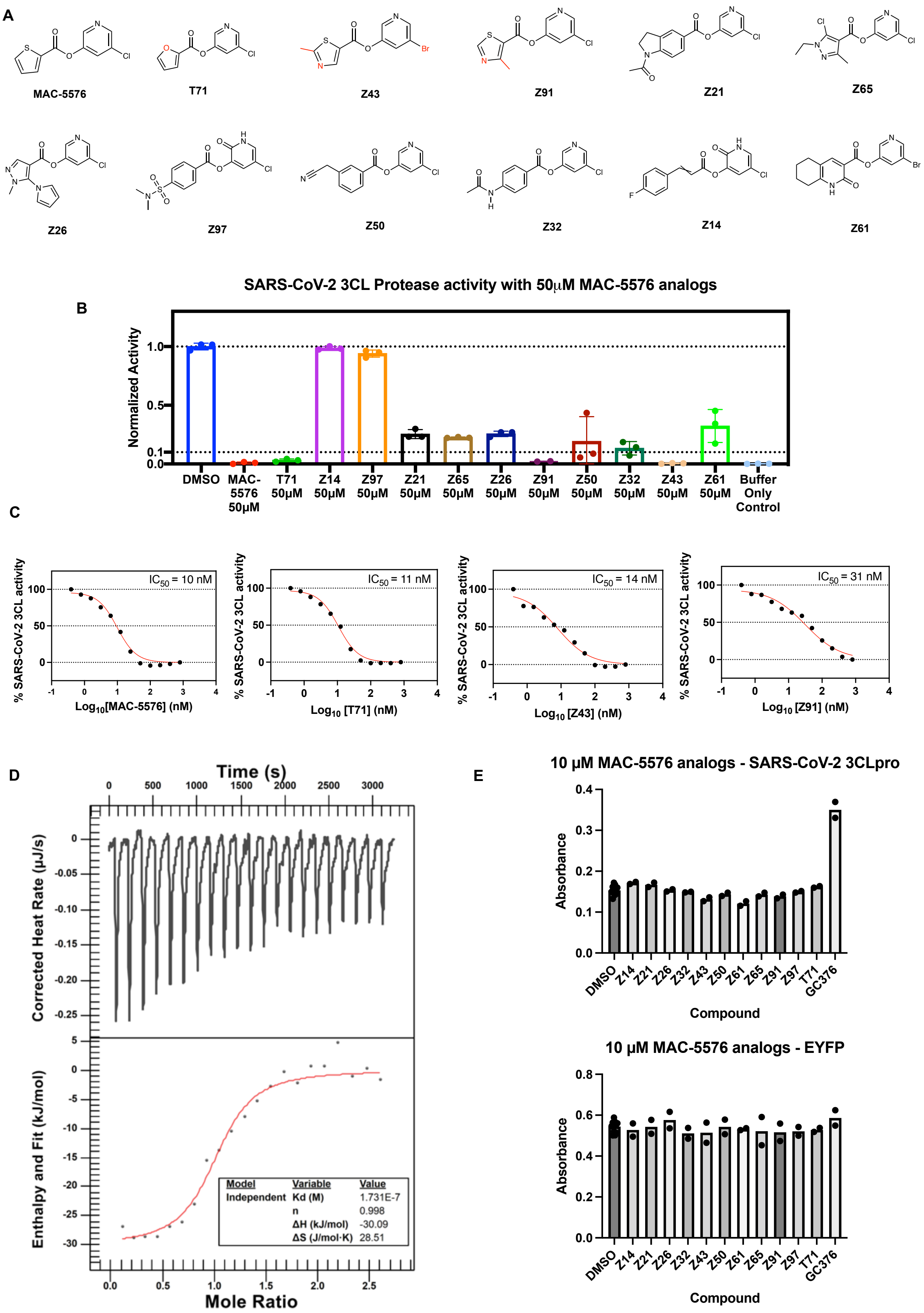
A
MAC-5576
T71
Z43
Z91
Z21
Z65
Z26
Z97
Z50
Z32
Z14
Z61
B
SARS-CoV-2 3CL Protease activity with 50µM MAC-5576 analogs
Normalized Activity
DMSO
MAC-5576 50µM
T71 50µM
Z14 50µM
Z97 50µM
Z21 50µM
Z65 50µM
Z26 50µM
Z91 50µM
Z50 50µM
Z32 50µM
Z43 50µM
Z61 50µM
Buffer Only Control
C
% SARS-CoV-2 3CL activity
IC50 = 10 nM
Log10[MAC-5576] (nM)
IC50 = 11 nM
Log10 [T71] (nM)
IC50 = 14 nM
Log10 [Z43] (nM)
IC50 = 31 nM
Log10 [Z91] (nM)
D
Time (s)
Corrected Heat Rate (µJ/s)
Enthalpy and Fit (kJ/mol)
Mole Ratio
Model
Variable
Value
Independent
Kd (M)
1.731E-7
n
0.998
ΔH (kJ/mol)
-30.09
ΔS (J/mol·K)
28.51
E
10 µM MAC-5576 analogs - SARS-CoV-2 3CLpro
Absorbance
Compound
10 µM MAC-5576 analogs - EYFP
DMSO
Z14
Z21
Z26
Z32
Z43
Z50
Z61
Z65
Z91
Z97
T71
GC376

Supplementary Fig. 10. Evaluation of MAC-5576 analogs as SRAS-CoV-2 3CL protease inhibitors.

A, Structures of MAC-5576 and analogs. **B**, in vitro activity of SARS-CoV-2 3CL protease treated with DMSO or 50μM MAC-5576 analogs. Experiments were performed in triplicate. Data are plotted as the mean ± s.d., n = 3 biological duplicates. **C**, The dose-dependent effects of MAC-5576, T71, Z43, and Z91 on activity of 20 nM SARS-CoV-2 3CL protease were tested. Mean of data were plotted, n = 2 biological duplicates. **D**, Representative ITC analysis of the in vitro binding of Z43 to SARS-CoV-2 3CL protease. Top: Raw titration data measured in μW. Each peak corresponds to a single 2.0 μL injection of 50 μM Z43 titrated into 5 μM 3CL-protease in 50 mM Tris Buffer, 1mM EDTA with 1% glycerol and 1.0% DSMO. Bottom: Area under each peak plotted against mole ratio and fitted to a one-site model. Each ligand was tested in duplicate. The average value along with standard error was reported in the text. The appropriate negative control of small molecule ligand into the running buffer was also performed. **E**, Evaluations of MAC-5576 analogs in a cell-based SARS-CoV-2 3CL protease inhibitor assay. Expression of SARS-CoV-2 3CL protease suppressed the viability of the 293T cells. Inhibitors of the protease rescue cell growth and boost the amount of crystal violet cell staining which is read on an absorbance plate reader. Cells expressing EYFP were included as control. Experiments were performed in duplicate. Mean of data were plotted as bar charts with an overlay of corresponding data points as dot plots, n = 2 biological replicates. Source data are provided as a Source Data file.

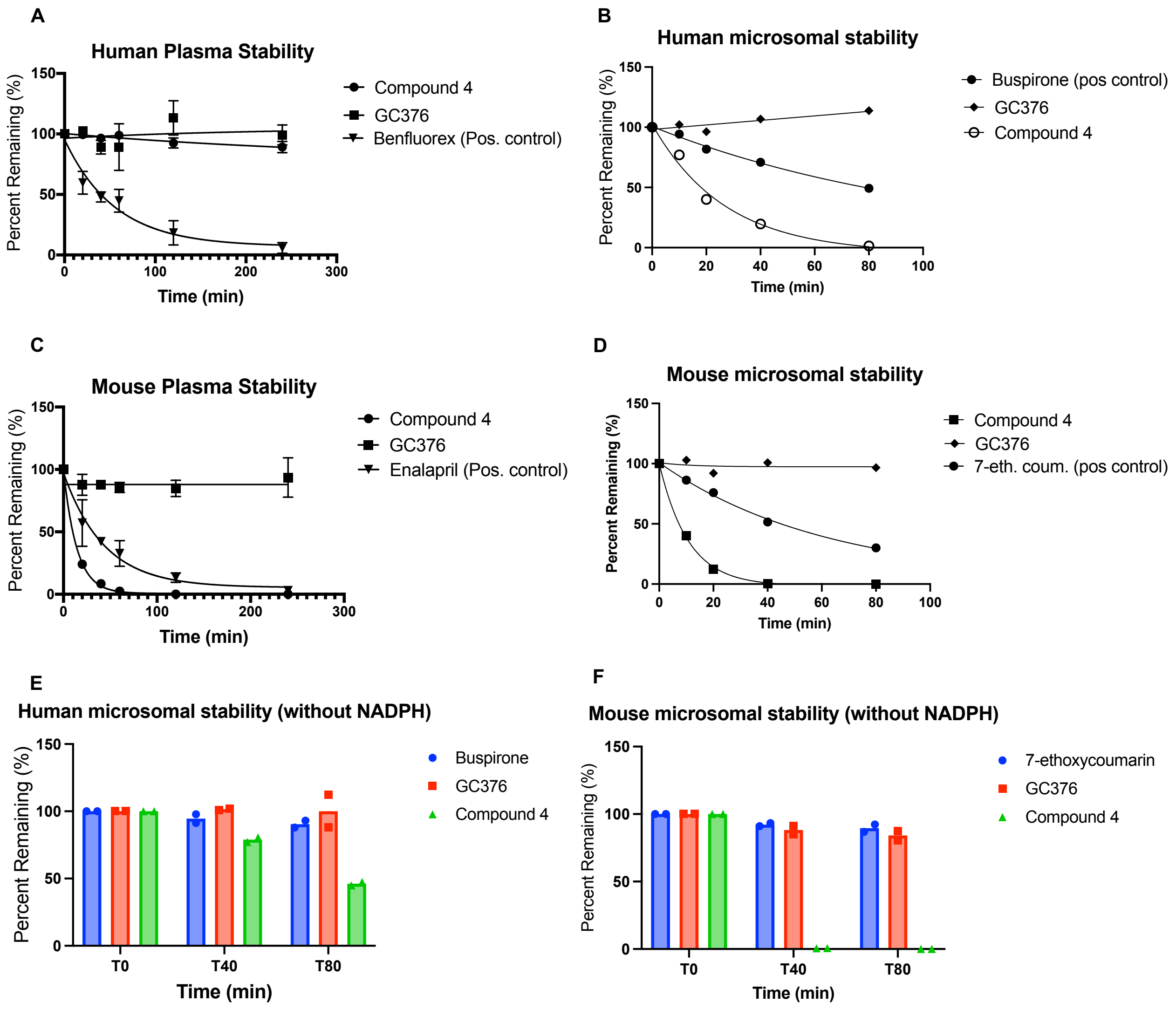

Supplementary Fig. 11. Metabolic stability of compound 4 and GC376. A, Human plasma stability of compound 4 and GC376. Data are plotted as the mean ± s.d., n = 3 biological replicates. **B**, Human microsome stability of compound 4 and GC376. Mean of data were plotted, n = 2 biological replicates. **C,** Mouse plasma stability of compound 4 and GC376. Data are plotted as the mean ± s.d., n = 3 biological replicates. **D**, Mouse microsomal stability of compound 4 and GC376. Mean of data were plotted, n = 2 biological replicates. **E,F**, Human and mouse microsomal stability of GC376 and compound 4 without NADPH. Mean of data were plotted as bar charts with an overlay of corresponding data points as dot plots, n = 2 biological replicates. Source data are provided as a Source Data file.

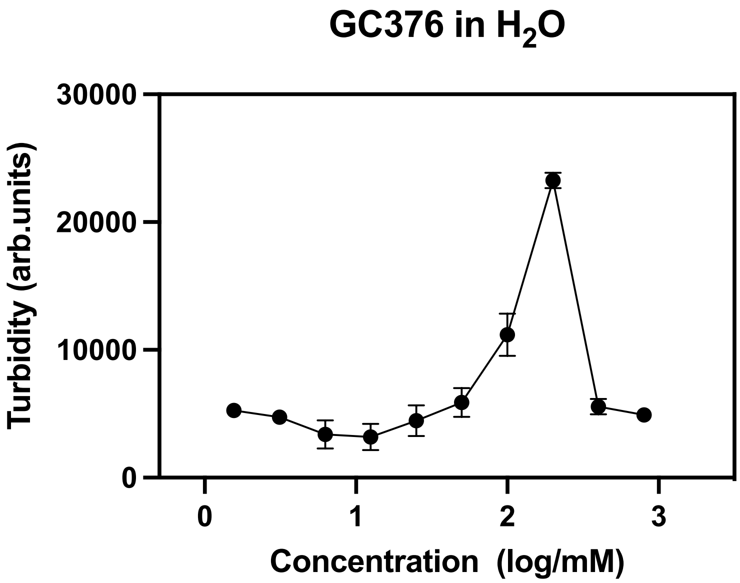

Supplementary Fig. 12. Solubility test (light scattering) of GC376 in water. Data are plotted as the mean ± s.d., n=3 biological replicates. Source data are provided as a Source Data file.

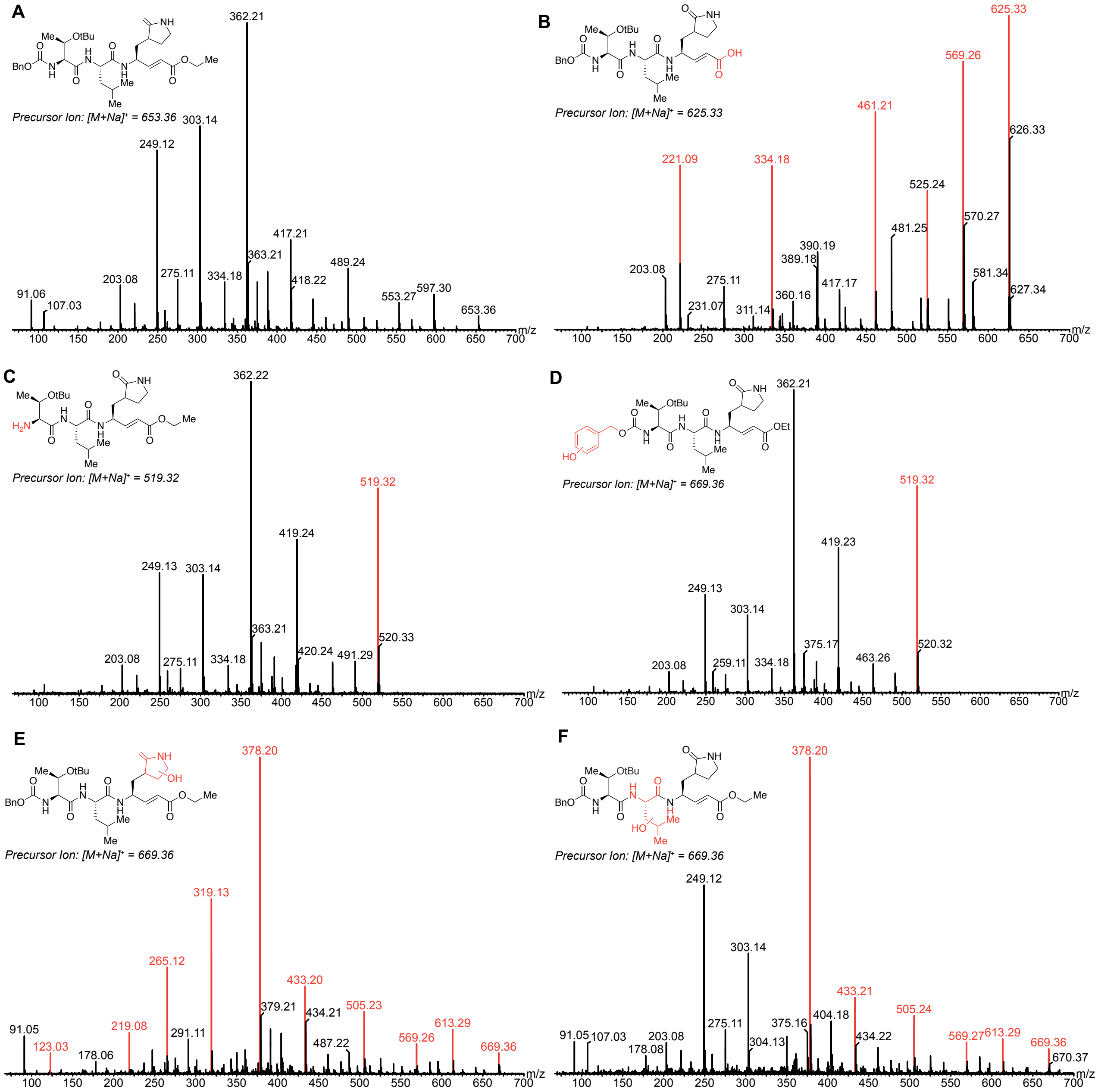

Supplementary Fig. 13. Identification of compound 4 metabolites by LC-MS/MS. Structures and MS/MS spectra of compound 4 (A) and proposed metabolites (B-F). Positions of structure modifications are highlighted in red. MS/MS product ion peaks that are consistent with these modifications are also highlighted in red.

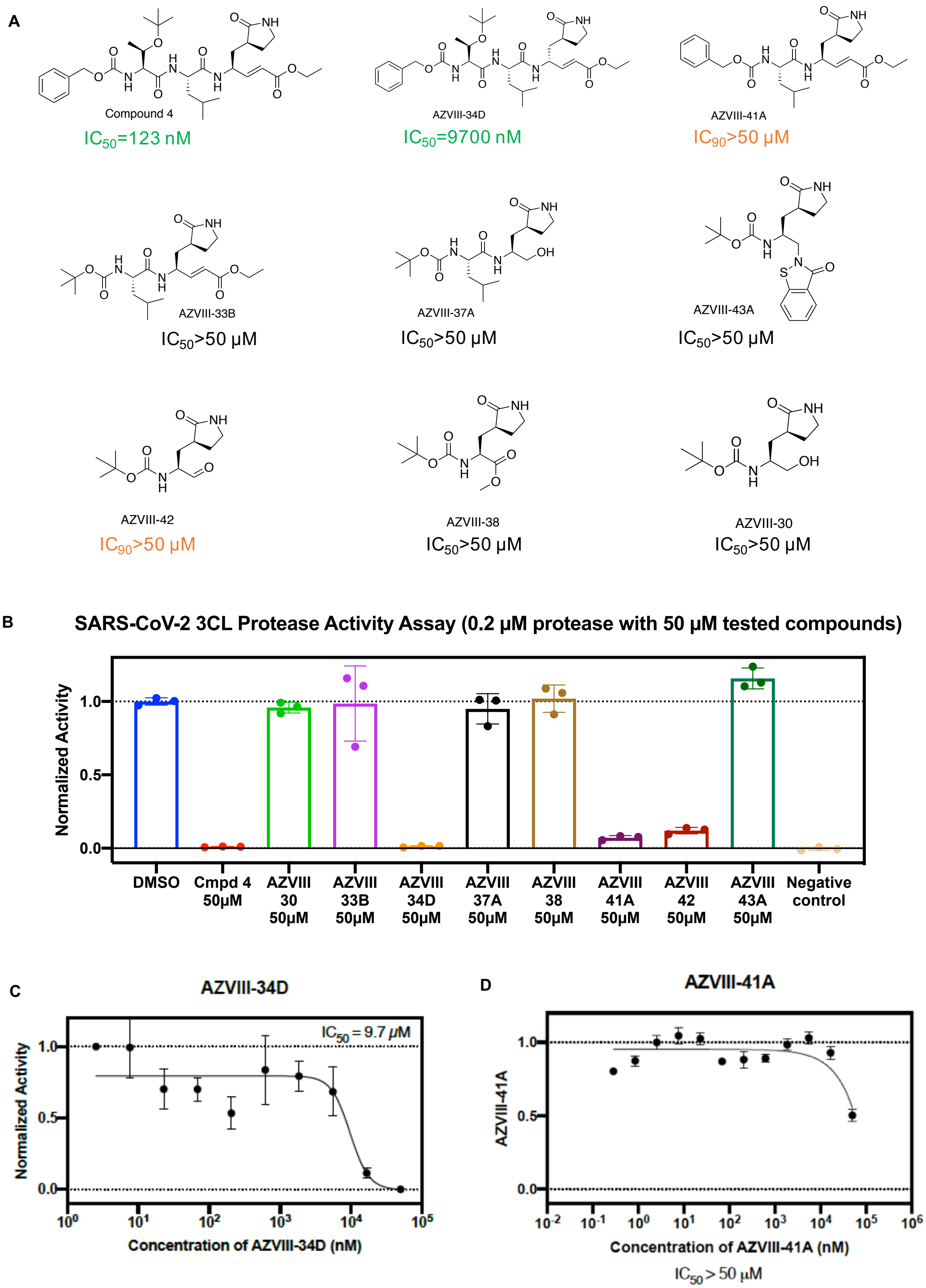
A
Compound 4
IC50=123 nM
AZVIII-34D
IC50=9700 nM
AZVIII-41A
IC90>50 μM
AZVIII-33B
IC50>50 μM
AZVIII-37A
IC50>50 μM
AZVIII-43A
IC50>50 μM
AZVIII-42
IC90>50 μM
AZVIII-38
IC50>50 μM
AZVIII-30
IC50>50 μM
B
SARS-CoV-2 3CL Protease Activity Assay (0.2 μM protease with 50 μM tested compounds)
Normalized Activity
DMSO
Cmpd 4 50μM
AZVIII 30 50μM
AZVIII 33B 50μM
AZVIII 34D 50μM
AZVIII 37A 50μM
AZVIII 38 50μM
AZVIII 41A 50μM
AZVIII 42 50μM
AZVIII 43A 50μM
Negative control
C
AZVIII-34D
IC50 = 9.7 μM
Normalized Activity
Concentration of AZVIII-34D (nM)
D
AZVIII-41A
AZVIII-41A
Concentration of AZVIII-41A (nM)
IC50 > 50 μM

Supplementary Fig. 14. Evaluation of compound 4 analogs as SRAS-CoV-2 3CL protease inhibitors.

A, Structures of compound 4 and analogs. **B**, in vitro activity of SARS-CoV-2 3CL protease treated with DMSO or 50μM compound 4 analogs. Experiments were performed in triplicate. Data are plotted as the mean ± s.d., n = 3 biological replicates . **C**, The dose-dependent effects of AZVIII-34D on the activity of 200nM SARS-CoV-2 3CL protease were tested. Data are plotted as the mean ± s.d., n = 3 biological replicates. **D**, The dose-dependent effects of AZVIII-41A on the activity of 200nM SARS-CoV-2 3CL protease were tested. Data are plotted as the mean ± s.d., n = 3 biological replicates. Source data are provided as a Source Data file.

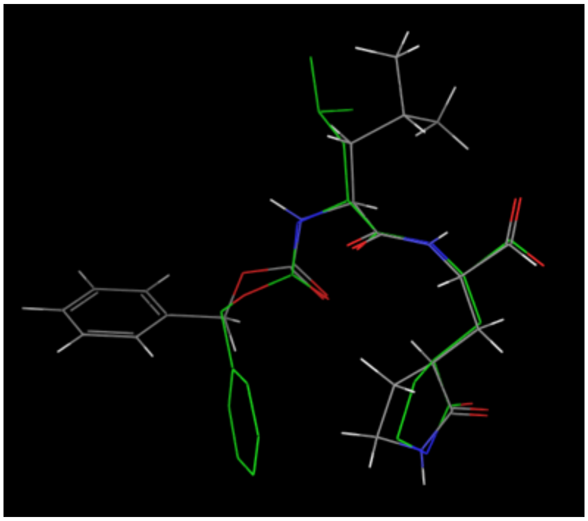

Supplementary Fig. 15. Overlay of a computer generated low energy conformation of GC376 (white) with its protein crystal bound conformation (colored) as revealed by X-ray.

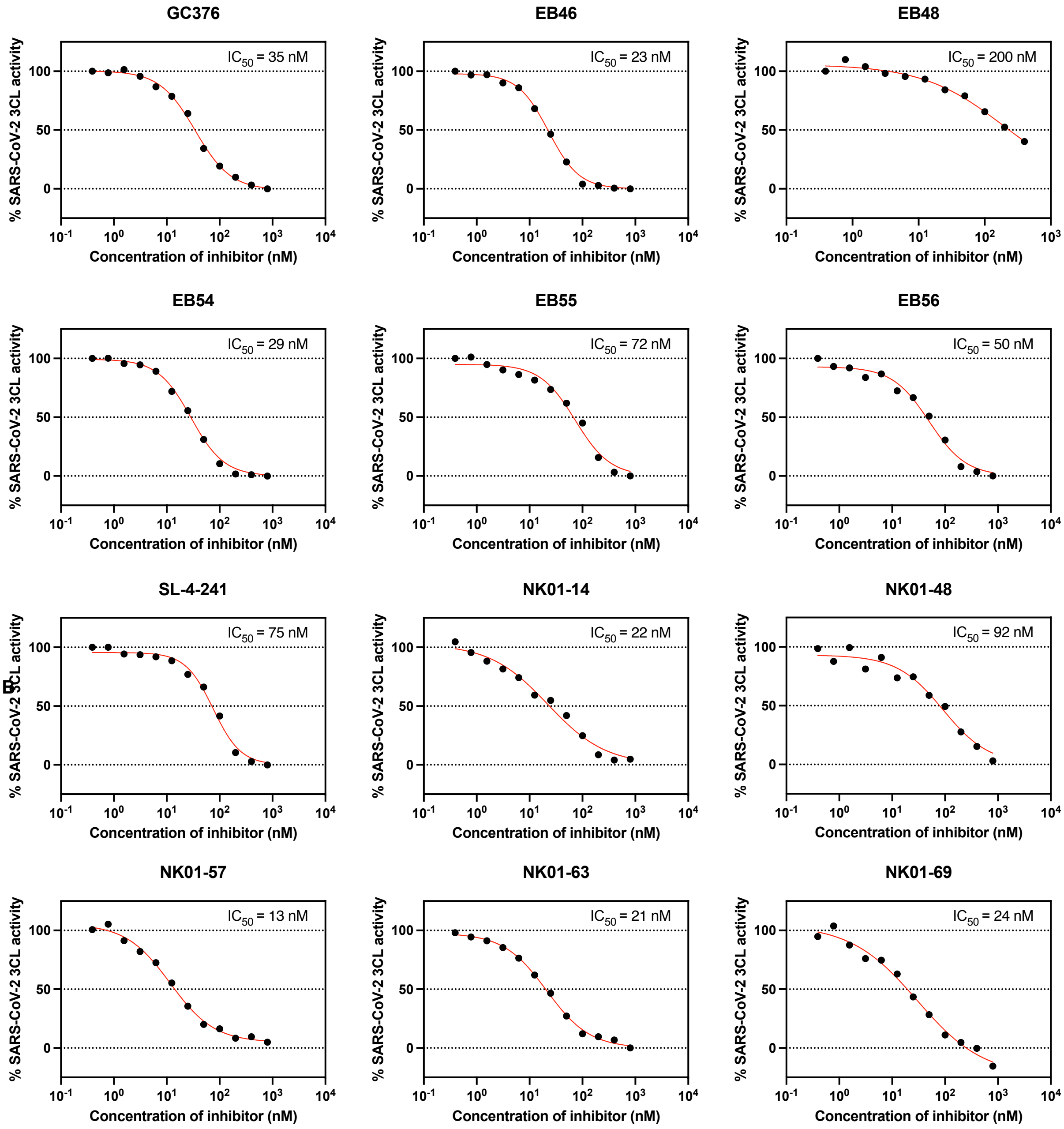

Supplementary Fig. 16. Evaluation of GC376 analogs as SARS-CoV-2 3CL protease inhibitor in biochemical assay. Measurement of the IC_{50} values for GC376 and its analogs on the activity of 20 nM SARS-CoV-2 3CL protease. Mean of data were plotted, n = 2 biological replicates. Source data are provided as a Source Data file.

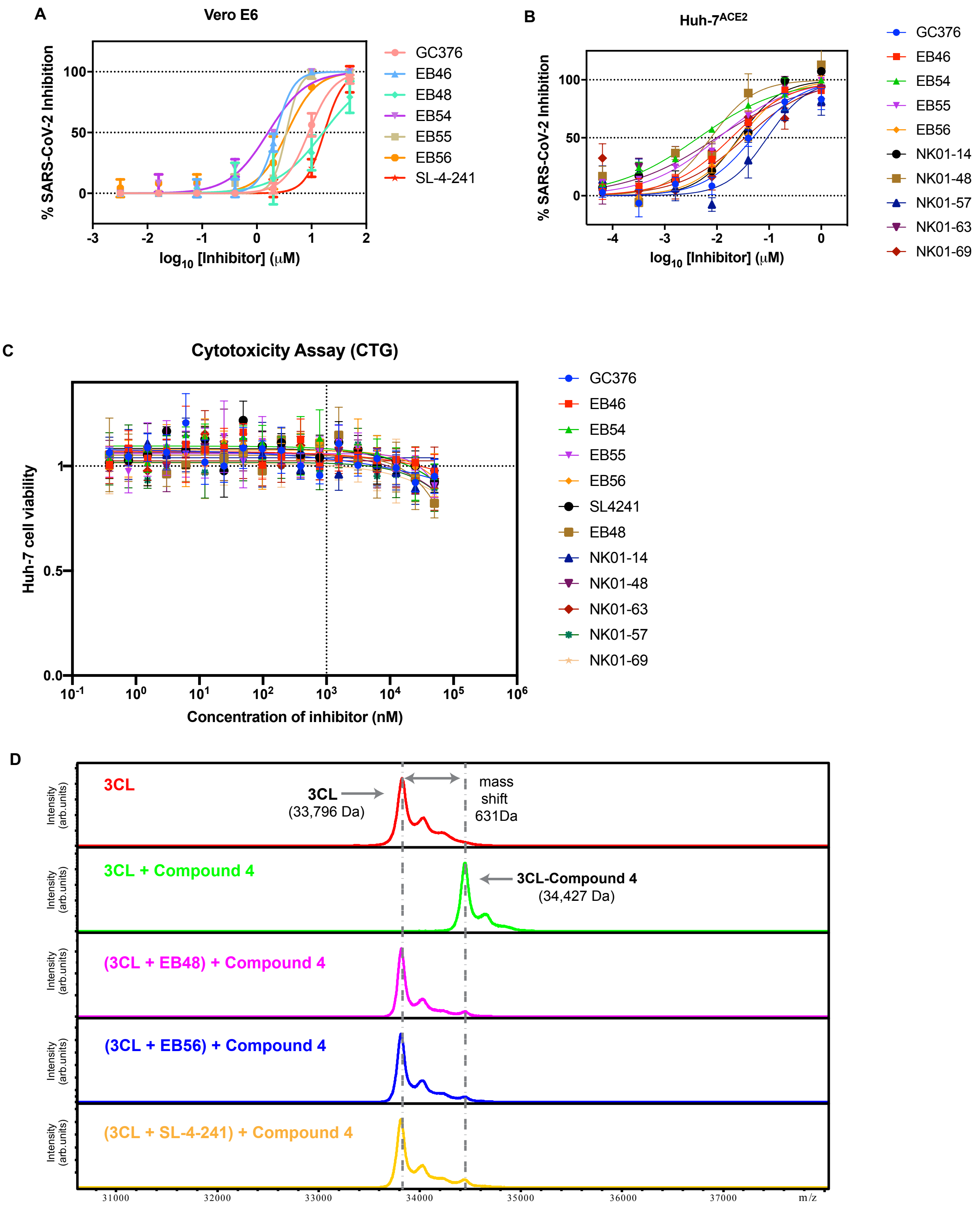

A
Vero E6
% SARS-CoV-2 Inhibition
log10 [Inhibitor] (μM)
GC376
EB46
EB48
EB54
EB55
EB56
SL-4-241
B
Huh-7ACE2
% SARS-CoV-2 Inhibition
log10 [Inhibitor] (μM)
GC376
EB46
EB54
EB55
EB56
NK01-14
NK01-48
NK01-57
NK01-63
NK01-69
C
Cytotoxicity Assay (CTG)
Huh-7 cell viability
Concentration of inhibitor (nM)
GC376
EB46
EB54
EB55
EB56
SL4241
EB48
NK01-14
NK01-48
NK01-63
NK01-57
NK01-69
D
3CL
3CL
(33,796 Da)
mass shift 631Da
3CL + Compound 4
3CL-Compound 4
(34,427 Da)
(3CL + EB48) + Compound 4
(3CL + EB56) + Compound 4
(3CL + SL-4-241) + Compound 4
Intensity (arb.units)
m/z

Supplementary Fig. 17. Evaluation of GC376 analogs as SARS-CoV-2 3CL protease inhibitor.

A, Ability of GC376 analogs to inhibit SARS-CoV-2 viral infection in Vero E6 cells. Stocks of SARS-CoV-2 strain 2019-nCoV/USA_WA1/2020 was propagated and titered in Vero-E6 cells. Serial dilutions of the test compound were prepared in cell media (EMEM + 10% FCS + penicillin/streptomycin), overlaid onto cells, and then virus was added to each well at an MOI of 0.2. Data are plotted as the mean ± s.d., n = 3 biological replicates. **B**, Ability of GC376 analogs to inhibit SARS-CoV-2 viral infection in Huh-7^{ACE2} cells. 0.05 MOI of SARS-CoV-2-mNeonGreen (a fluorescent reporter variant of strain 2019-nCoV/USA_WA1/2020) and serial dilutions of the test compound were added to the cells. Data are plotted as the mean ± s.d., n = 3 biological replicates. **C**, Cytotoxicity test of GC376 analogs in Huh-7^{ACE2} cells. Data are normalized to DMSO control and plotted as the mean ± s.d., n = 3 biological replicates. **D**, Intact protein MALDI MS of 3CL only (red), 3CL treated with 10 eq compound 4 for 1 hour at 4˚C (green), 3CL preincuated with 1 eq EB48 (pink), EB56 (blue), or SL-4-241 (yellow) for 1 hour at 4˚C before buffer exchange and sequential treatment of 10 eq compound 4 for 1 hour at 4˚C. Source data are provided as a Source Data file.

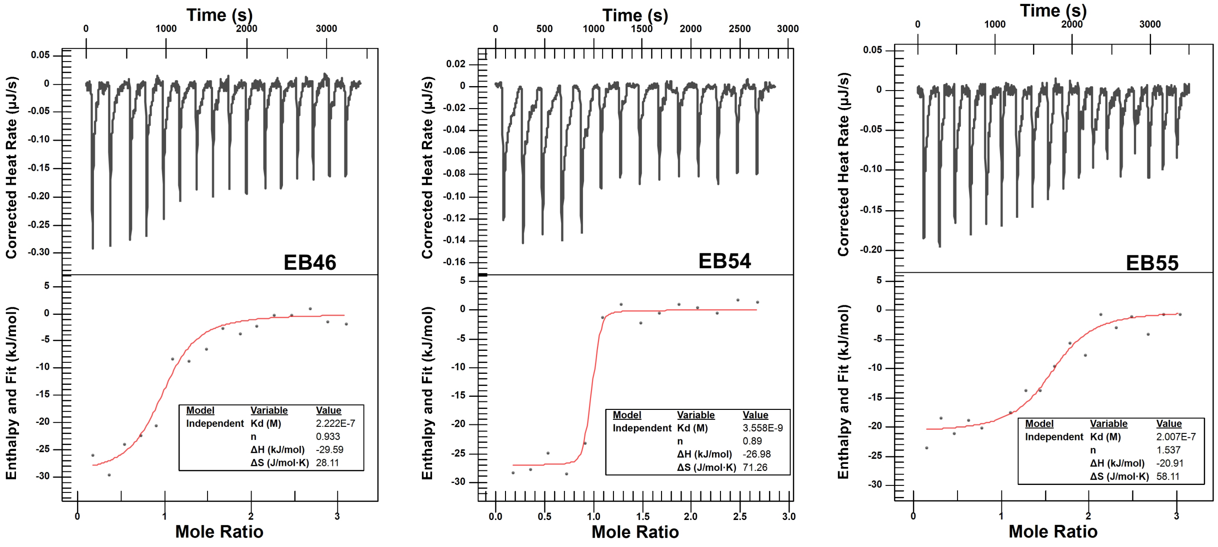

Supplementary Fig. 18. Representative ITC analysis of the in vitro binding of EB46, EB54, and EB55 to SARS-CoV-2 3CL protease. Top: Raw titration data measured in μW. Each peak corresponds to a single 2.0 μL injection of 50 μM compound titrated into 5 μM 3CL-protease in 50 mM Tris Buffer, 1mM EDTA with 1% glycerol and 1.0% DSMO. Bottom: Area under each peak plotted against mole ratio and fitted to a one-site model. Each ligand was tested in biological duplicates. The average value along with standard error was reported in the text. The appropriate negative control of small molecule ligand into the running buffer was also performed.

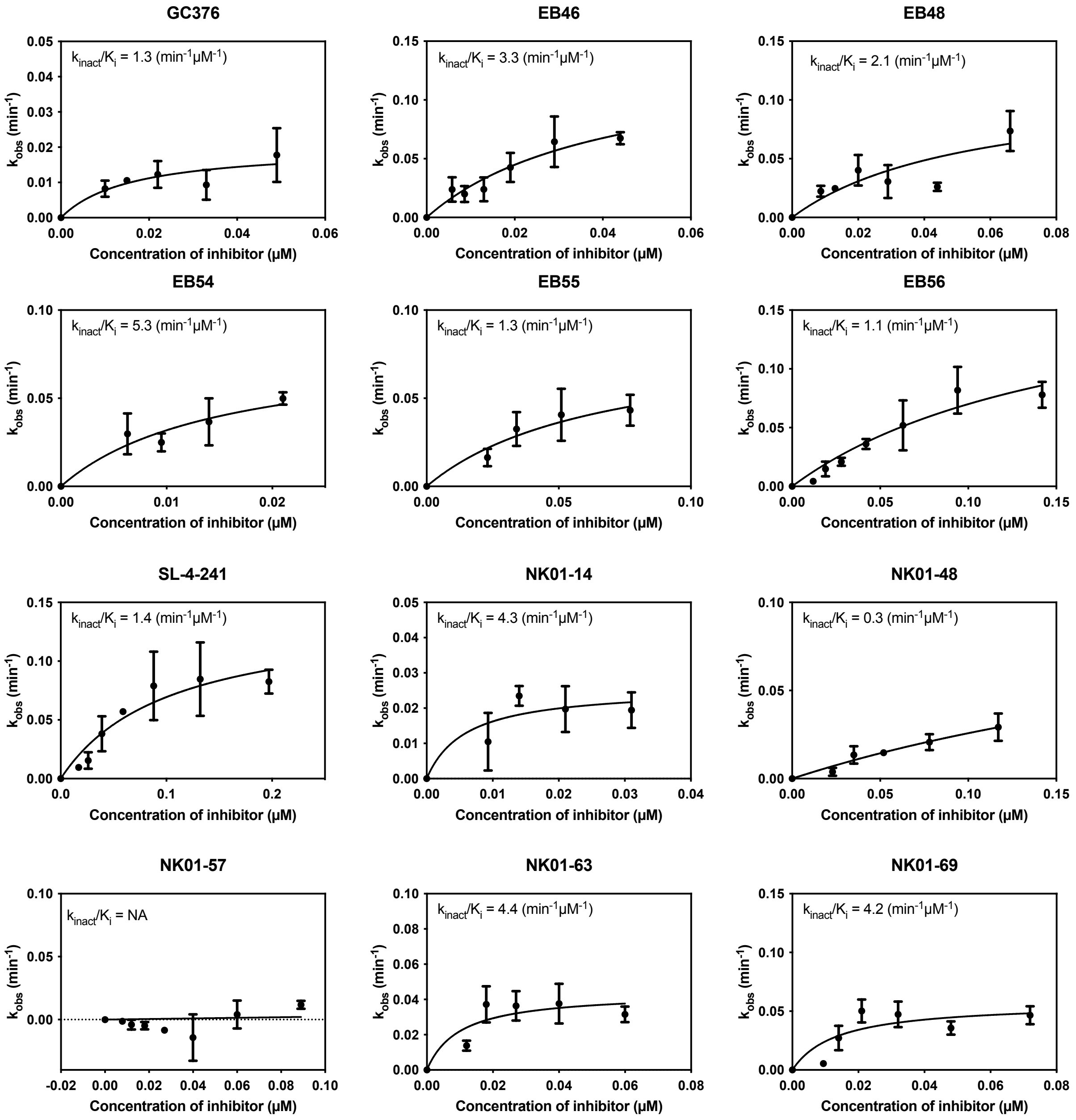

Supplementary Fig. 19. Evaluation of GC376 analogs as SARS-CoV-2 3CL protease inhibitor in kinetic enzymatic assay. Determination of the k_{inact}/K_I values for GC376 and its analogs on the activity of 25 nM SARS-CoV-2 3CL protease. Data are plotted as the mean ± s.d., n = 3 biological replicates. Source data are provided as a Source Data file.

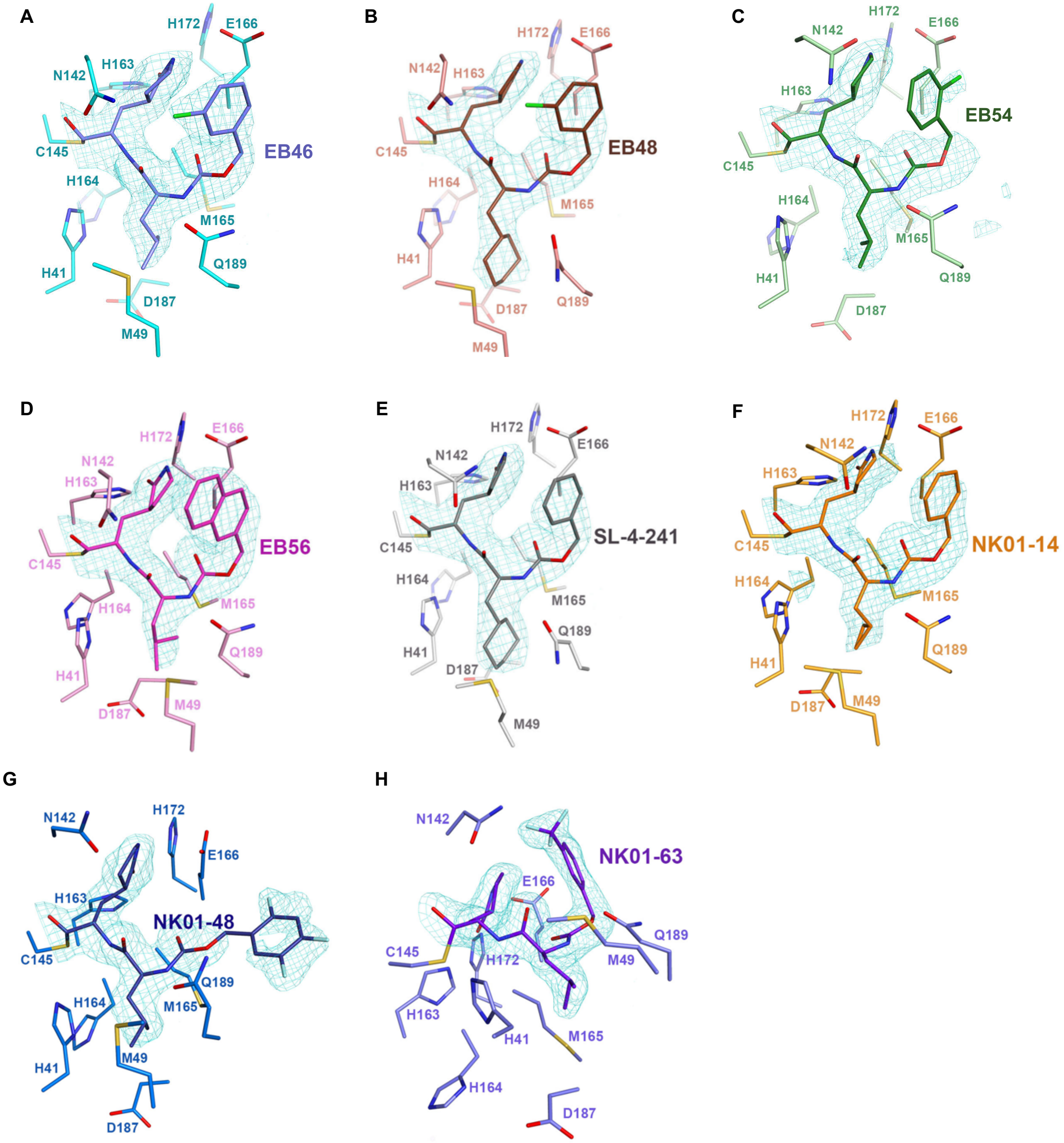

Supplementary Fig. 20. 3CL active site residues interacting with 8 different inhibitors. 3CL active site shown in complex with inhibitor EB46 (A), EB48 (B), EB54 (C), EB56 (D), SL-4-241 (E), NK01-14 (F), NK01-48 (G), and NK01-63 (H) in the corresponding co-crystal structures. Inhibitors and the side chain of residues interacting

with the inhibitor are depicted with stick models. Fo-Fc omit map contoured at 3 sigma is also shown for all inhibitors.

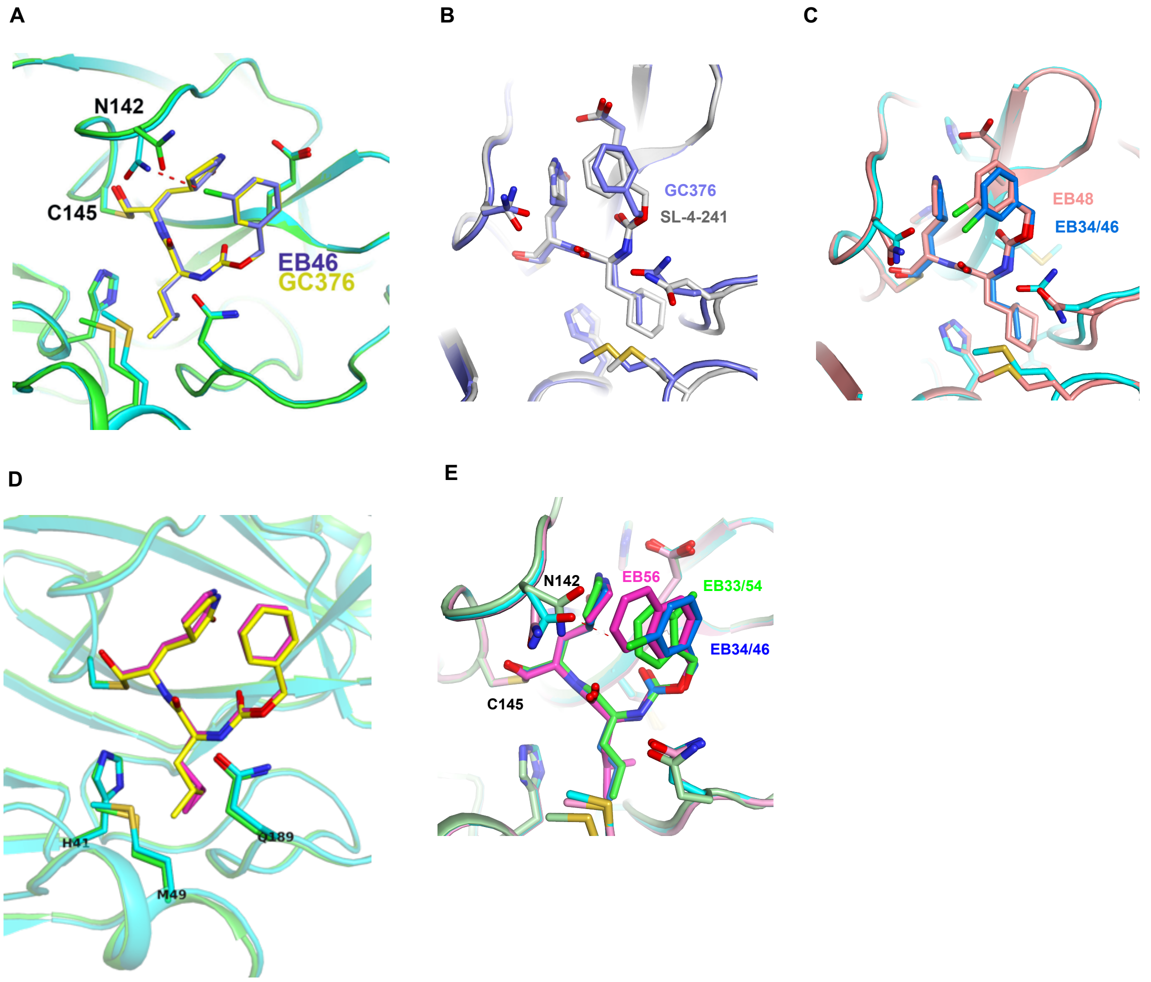

Supplementary Fig. 21. Overlap analysis for co-crystal structures of 3CL with GC376 analogs.

A, The co-crystal structure of 3CL(cyan)-EB46(purple) complex, overlaid with 3CL (green)-GC376(yellow) complex, revealed that the Cl group of EB46 made an additional H-bond with Asn-142 of SARS-CoV-2 3CL protease. **B,** Alignment of the co-crystal structure of 3CL-SL-4-241 and 3CL-GC376 complex. **C,** Alignment of the co-crystal structure of 3CL-EB48 with 3CL-EB46 (= 3CL-EB34). **D**, Structural overlay of 3CL-GC376 and 3CL-NK01-14. GC376 and NK01-14 are shown in yellow and light magenta and the

respective 3CL protease in green and cyan. **E,** Alignment of the co-crystal structure of 3CL-EB54 (=3CL-EB33), 3CL-EB46 (= 3CL-EB34), and 3CL-EB56 complex.

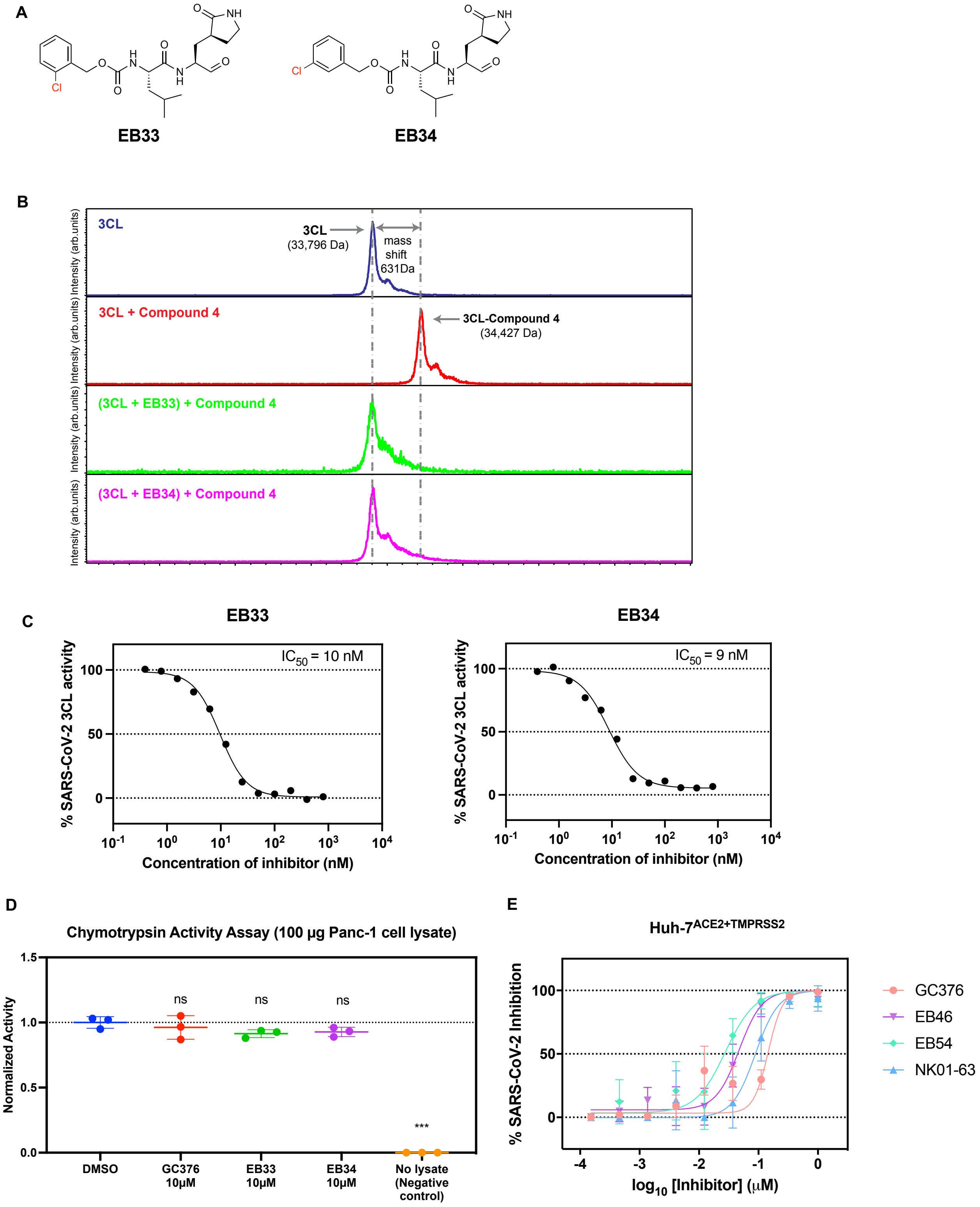
A
EB33
EB34
B
3CL
3CL (33,796 Da)
mass shift 631Da
3CL + Compound 4
3CL-Compound 4 (34,427 Da)
(3CL + EB33) + Compound 4
(3CL + EB34) + Compound 4
Intensity (arb.units)
C
EB33
IC50 = 10 nM
EB34
IC50 = 9 nM
% SARS-CoV-2 3CL activity
Concentration of inhibitor (nM)
D
Chymotrypsin Activity Assay (100 µg Panc-1 cell lysate)
Normalized Activity
ns
***
DMSO
GC376 10µM
EB33 10µM
EB34 10µM
No lysate (Negative control)
E
Huh-7ACE2+TMPRSS2
% SARS-CoV-2 Inhibition
log10 [Inhibitor] (µM)
GC376
EB46
EB54
NK01-63

Supplementary Fig. 22. Characterization of GC376 aldehyde analogs EB33 and EB34 and the selectivity of GC376 analogs towards SARS-CoV-2 3CL protease. A, Structures of EB33 and EB34. **B**, Intact protein MALDI MS of 3CL only (blue), 3CL treated with 10 eq compound 4 for 1 hour at 4˚C (red), 3CL preincuated with 1 eq EB33 for 1 hour at 4˚C before treatment of 10 eq compound 4 for 1 hour at 4˚C (green), and 3CL preincuated with 1 eq EB34 for 1 hour at 4˚C before treatment of 10 eq compound 4 for 1 hour at 4˚C (green). **C**, IC_{50} values of EB33 and EB34 on the in vitro SARS-CoV-2 3CL protease activity were tested. Experiments were performed in duplicate. Data are plotted as the mean ± s.d., n = 2 biological replicates. **D,** The in vitro biochemical activities of human chymotrypsin in the Panc-1 cell lysates and the biochemical activities of human Caspase-3 in the lysates of HT1080 cells (induced by staurosporine) were measured after incubation with DMSO, GC376, EB33, and EB34. Experiments were performed in triplicate. Data are plotted as the mean ± s.d., n = 3 biological replicates. Two-tailed t test was performed versus DMSO. P^{ns} of GC376 = 0.57, P^{ns} of EB33 = 0.13, P^{ns} of EB34 = 0.23, and P^{***} of no lysate = 0.0007, all versus DMSO control. **E**, Measurement of SARS-CoV-2 viral inhibition in Huh-$7^{ACE2+TMPRSS2}$ cells treated with GC376, EB46, EB54, or NK01-63. Experiments were performed in triplicate. Data are plotted as the mean ± s.d., n = 3. Source data are provided as a Source Data file.

Supplementary Table 1. Evaluation of EB54 and NK01-63 with a panel of proteases.

Protease	Protease Source	Protease Class	EB54 IC_{50} (μM)	NK01-63 IC_{50} (μM)
3CL	SARS-CoV-2 recombinant	Cysteine	0.029	0.016
ACE1	Human recombinant	dipeptidase	>100	>100
ACE2	Human recombinant	dipeptidase	>100	>100
Caspase 8	Human recombinant	Cysteine	52	18
Cathepsin B	Human liver	Cysteine	0.35	1.1
Cathepsin K	Human recombinant	Cysteine	0.02	0.04
Cathepsin S	Human recombinant	Cysteine	0.006	0.03
Cathepsin L (substrate 1)	Human liver	Cysteine	0.001	0.006
Cathepsin L (substrate 2)	Human liver	Cysteine	0.006	0.17
Elastase	Human Neutrophil	Serine	>100	>100
Factor Xa	Human plasma	Serine	>100	>100
Furin	Human recombinant	Serine	>100	>100
MMP 1	Human recombinant	metalloproteinase	>100	>100
PLpro	SARS-CoV-2 recombinant	Cysteine	>100	>100
Thrombin a	Human plasma	Serine	>100	>100
TMPRSS2	Human recombinant	Serine	>100	>100
Trypsin	Bovine pancreas	Serine	>100	>100
Urokinase	Human urine	Serine	>100	>100

.

Supplementary Table 2. Comparison of NK01-63 with current benchmark SARS-CoV-2 3CL^{pro} inhibitors.

Compound	SARS-CoV-2 3CL^{pro} IC_{50} (nM)	SARS-CoV-2 antiviral EC_{50} (nM)	Pan-Coronavirus Activity	Resolution of co-crystal structure with SARS-CoV-2 3CL^{pro} (Å)	Ref.
GC376	42	51 (Huh-7^{ACE2})	MERS, SARS	1.94	Nature Comm. 2021[1]
NK01-63	6	6 (Huh-7^{ACE2})	MERS, SARS, HCoV-229E, HCoV-OC43	1.57	
PF-00835231 (Intravenous)	N/A*	221 (A549^{ACE2})	SARS, HCoV-229E	1.41	J Med Chem 2020[2] JVI 2021[3]
PF-07321332 (Oral)	N/A*	78 (A549^{ACE2})	MERS, SARS, HCoV-229E	1.91	Science 2021[4]
2c	70**	190 (Vero+CP***)	N/A	N/A	Eur J Med Chem. 2021[5]
2d	80**	180 (Vero+CP***)	N/A	2.0	Eur J Med Chem. 2021[5]
MI-09	15	35 (Huh-7^{ACE2})	N/A	N/A	Science 2021[6]
MI-30	17	31 (Huh-7^{ACE2})	N/A	N/A	Science 2021[6]
Compd. 7	260	11,000 (Vero E6)	N/A	2.35	Nature Comm. 2021[7]
ALG-097111	7	200 (A549^{ACE2})	HCoV-229E, HCoV-OC43	N/A	Biochem. Biophys. Res. Commun. 2021[8]
Calpeptin	N/A*	72 (Vero E6)	N/A	2.50	Science 2021[9]

* : IC50 values of PF-00835231, PF-07321332, and Calpeptin were not reported in literature.
** : In the same dataset reported by reference, IC_{50} of GC376 is 190 nM.
*** : Vero cells featured high efflux of GC376 analogs, so efflux inhibitor CP was applied.

Supplementary Table 3. Assay conditions for protease panel.

Protease	Enzyme Source	Substrate	Substrate conc.(μM)	Ex/Em (nm)	Buffer	Control Inhibitor
ACE1	Human recombinant aa30-1261	MCA-RPPGFSAFK(Dnp)-OH	10	320/405	Buffer A	Captopril
ACE2	Human recombinant aa18-740	MCA-YVADAPK(Dnp)-OH	10	320/405	ACE2 Buffer	ACE2 Inhibitor
Caspase 8	Human recombinant aa217-479	Ac-LEHD-AMC	5	355/460	Caspase buffer 2	IETD-CHO
Cathepsin B	Human liver	Z-FR-AMC	10	355/460	Buffer B'	E64
Cathepsin L (Substrate 1)	Human liver	Z-FR-AMC	10	355/460	Buffer L	E64
Cathepsin L (Substrate 2)	Human liver	MCA-PLGL-Dap(Dnp)-AR-NH2	10	355/460	Buffer L	E64
Elastase	Human Neutrophil	MeOSuc-AAPV-AMC	10	355/460	Elastase Buffer	Sivelestat
Factor Xa	Human plasma	CH_3SO_2-D-CHA-Gly-Arg-AMC-AcOH	10	355/460	Buffer A +0.25mg/ml BSA	Gabexate mesylate (GM)
Furin	Human recombinant aa108-715	pERTKR-AMC	5	355/460	Furin buffer	Decanoyl-RVKR-CMK
MMP1	Human recombinant aa81-249	(5-FAM/QXLTM) FRET peptide [QXL520-g-Abu-P-Cha-Abu-Smc-H-A-Dab(5-FAM)-A-K-NH2]	5	485/520	MMP Buffer	GM6001
SARS-CoV-2 PLpro	recombinant	Z-RLRGG-AMC	10	355/460	Buffer B +EDTA	PLpro inhibitor
Thrombin alpha	Human plasma	H-D-CHA -Ala-Arg-AMC.2AcOH	10	355/460	Buffer A + 2.5 mM CaCl2 + 1 mg/ml BSA	Gabexate mesylate (GM)
TMPRSS2	Human recombinant aa106-492	Boc-Gln-Ala-Arg-AMC	25	355/460	Buffer A'	Camostat
Trypsin	Bovine pancreas	H-D-CHA -Ala-Arg-AMC.2AcOH	10	355/460	Buffer A	Gabexate mesylate (GM)
Urokinase	Human urine	Bz-b-Ala-Gly-Arg-AMC.AcOH	10	355/460	Buffer A	Gabexate mesylate (GM)

Supplementary Table 4. Crystallography data collection and refinement statistics for EB46, EB48, EB54, and EB56.

**Protein**	3CL M^{pro}	3CL M^{pro}	3CL M^{pro}	3CL M^{pro}
**Inhibitor**	EB46	EB48	EB54	EB56
Space group	*C*2	*C*2	*C*2	*C*2
Cell dimensions				
a, *b*, *c* (Å)	97.2, 81.0, 54.5	96.4, 81.5, 54.6	97.3, 81.6, 51.8	96.7, 81.5, 54.7
α, β, γ (°)	90, 116.4, 90	90, 116.5, 90	90, 114.5, 90	90, 116.6, 90
Resolution (Å)	48.81-1.56 (1.58-1.56)*	59.23-1.88 (1.88-1.92)*	47.11-1.60 (1.63-1.60)*	59.30-1.91 (1.96-1.91)*
R_{merge} (%)	4.3 (115.6)	25.6 (234.7)	5.9 (158.9)	14.5 (132.9)
$I/\sigma I$	17.6 (1.1)	9.0 (0.7)	14.4 (0.9)	10.6 (1.0)
Completeness (%)	98.2 (83.2)	98.5 (81.9)	98.5 (82.6)	98.1 (80.4)
Redundancy	6.7 (4.8)	6.9 (6.3)	6.9 (6.1)	6.9 (5.4)
CC1/2	1.00 (0.74)	0.99 (0.43)	1.00 (0.67)	1.00 (0.61)
**Refinement**				
Resolution (Å)	48.81-1.65 (1.67-1.65)*	48.90-2.08 (2.13-2.08)	47.11-1.68 (1.70-1.68)*	47.07-2.03 (2.08-2.03)
No. reflections	45,001 (4,487)	22,682 (2,246)	41,660 (4,177)	25,427 (2,556)
R_{work} / R_{free} (%)	16.8 (27.9)/ 19.6 (31.7)	17.6 (31.7)/ 21.6 (40.8)	17.4 (26.9)/ 19.8 (28.2)	17.2 (27.6)/ 21.5 (33.9)
Ramachandran Plot (%)				
Outliers	0.00	0.00	0.00	0.00
Allowed	0.67	2.68	1.99	2.00
Favored	99.33	97.32	98.10	98.00
No. atoms				
Protein	2,329	2,329	2,340	2,333
Ligand/ion	35	33	34	33
Water	193	133	166	138
B-factors				
Protein	47.8	48.5	50.3	48.7
Ligand/ion	56.5	48.0	54.9	43.2
Water	54.5	52.0	54.9	49.8
R.m.s deviations				
Bond lengths (Å)	0.006	0.007	0.006	0.007
Bond angles (°)	0.8	0.8	0.8	0.8
PDB code	7TIU	7TIV	7TIW	7TIX

*Highest resolution shell is shown in parenthesis.

Supplementary Table 5. Crystallography data collection and refinement statistics for SL-4-241, NK01-14, NK01-48, and NK01-63.

**Protein**	3CL M^{pro}	3CL M^{pro}	3CL M^{pro}	3CL M^{pro}
**Inhibitor**	SL-4-241	NK01-14	NK01-48	NK01-63
Space group	*C*2	*C*2	*C*2	*C*2
Cell dimensions				
a, *b*, *c* (Å)	96.7, 81.5, 54.7	97.5, 81.8, 51.9	98.2, 80.5, 54.5	97.4, 81.6, 51.8
α, β, γ (°)	90, 116.6, 90	90, 114.6, 90	90, 116.5, 90	90, 114.5, 90
Resolution (Å)	59.15-1.95 (2.00-19.5)*	47.17-1.57 (1.59-1.57)*	59.36-1.64 (1.67-1.64)*	47.17-1.49 (1.51-1.49)*
R_{merge} (%)	18.2 (200.5)	5.0 (160.8)	11.7 (189.8)	5.6 (168.2)
$I/\sigma I$	7.3 (0.7)	15.5 (1.1)	9.6 (0.8)	16.7 (1.1)
Completeness (%)	98.1 (79.3)	98.1 (90.0)	1.0 (90.3)	97.8 (87.7)
Redundancy	6.8 (5.8)	6.9 (6.4)	6.8 (6.0)	6.8 (6.1)
CC1/2	0.99 (0.26)	1.00 (0.56)	1.00 (0.53)	1.00 (0.57)
**Refinement**				
Resolution (Å)	48.76-2.17 (2.22-2.17)	47.17-1.64 (1.66-1.64)*	59.36-1.79 (1.82-1.79)*	47.17-1.55 (1.57-1.55)*
No. reflections	19,875 (1,955)	44,624 (4,495)	35,542 (3,427)	52,603 (5,127)
R_{work} / R_{free} (%)	16.6 (22.3)/ 21.7 (26.4)	17.3 (26.1)/ 19.0 (25.2)	16.5 (24.3)/ 19.5 (31.6)	17.0 (27.9)/ 19.1 (29.4)
Ramachandran Plot (%)				
Outliers	0.00	0.00	0.00	0.00
Allowed	2.68	1.00	1.00	1.00
Favored	97.32	99.00	99.00	99.00
No. atoms				
Protein	2,329	2,340	2,334	2,340
Ligand/ion	32	30	46	38
Water	106	158	207	230
B-factors				
Protein	51.1	45.9	38.6	41.5
Ligand/ion	45.7	40.1	40.4	41.0
Water	42.5	49.2	46.4	49.8
R.m.s deviations				
Bond lengths (Å)	0.007	0.006	0.006	0.006
Bond angles (°)	0.8	0.8	0.8	0.8
PDB code	7TJ0	7TIA	7TIY	7TIZ

*Highest resolution shell is shown in parenthesis.

Supplementary Note 1. Synthesis of compounds

Unless noted, all the following reactions were performed in flame or oven-dried glassware and carried out under an atmosphere of argon or nitrogen with magnetic stirring, unless otherwise stated. Anhydrous solvents were used as supplied (Acros Organics ExtraDry or over molecular sieves). N,N-Diisopropylamine was purified by distillation from potassium hydroxide under argon. All other reagents were used as supplied unless otherwise stated (Aldrich, Chem-Impex, TCI America, etc). Thin layer chromatography was performed on SiliCycle® 250 μm 60 Å plates or Merck Kieselgel 60 F254 0.20 mm precoated, glass backed silica gel plates. Visualization was accomplished with 254 nm UV light, $KMnO_4$ stain, and/or by p-anisaldehyde staining solution. All column chromatography was performed using general flash techniques on SiliCycle® SilicaFlash® P60, 40-63 μm 60 Å. For particularly difficult separations, automated normal phase flash column chromatography was carried out on Teledyne Isco Combiflash RF Plus using CombiFlash gold pre-packed columns with UV/ELS detector using HPLC grade solvent (Fisher Scientific) with the indicated solvent system.

NMR spectra were recorded on Bruker AV III 400 or AV III 500 MHz spectrometers at ambient temperature. Chemical shifts (δ) for ^{1}H NMR spectra are reported in parts per million (ppm) from Me_4Si with the solvent resonance as the internal standard ($CDCl_3$ = 7.26 ppm, CD_3OD = 3.31 ppm) with multiplicity (*s* = singlet, *bs* = broad singlet, *d* = doublet, *t* = triplet, *q* = quartet, and *m* = multiplet) and coupling constants (in Hz). ^{13}C NMR spectra are reported in ppm from Me_4Si with the solvent resonance as the internal standard ($CDCl_3$ = 77.16 ppm, CD_3OD = 49.00 ppm). ^{19}F NMR spectra are reported in ppm from

CDCl3 and are uncorrected. High Resolution Mass spectrometry (HRMS) was carried out at the Mass Spectrometry Facility at the Chemistry Department of Columbia University in the City of New York and recorded on a Waters Acquity H UPLC-MS.

Preparation of 2-sufonylpyridine analogs

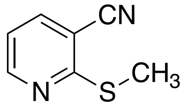

2-(methylthio)nicotinonitrile (NT-1-21) The title compound was prepared according to a published procedure[10]; spectral data are in agreement with literature values.

^{1}H NMR (500 MHz, $CDCl_3$) δ 8.60 (dd, J = 5.0, 1.8 Hz, 1H), 7.79 (dd, J = 7.7, 1.8 Hz, 1H), 7.07 (dd, J = 7.7, 4.9 Hz, 1H), 2.64 (s, 3H).

^{13}C NMR (126 MHz, $CDCl_3$) δ 163.7, 152.2, 140.6, 118.4, 115.7, 107.5, 13.4.

HRMS High accuracy (ASAP)**:** Calculated for $C_7H_6N_2S$ $(M+H)^+$: 151.0330; found: 151.0327.

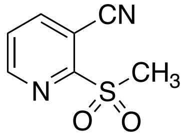

2-(methylsulfonyl)nicotinonitrile (NT-1-24) The title compound was prepared from **NT-1-21** according to a modified procedure from the literature[11].

Pyridine **NT-1-21** (87 mg, 0.58 mmol, 1 equiv) was weighed into 50 mL round bottom flask equipped with a stir bar. The solid was dissolved in 10 mL of anhydrous MeOH, followed by portion-wise (usually 3 portions) additions of mCPBA (500 mg, 2.9 mmol, 5 equiv). Substrate conversion was monitored via TLC analysis (70% EtOAc:Hex to 100% EtOAc), with up to an additional 5 equiv of mCPBA added if necessary. Upon complete conversion of starting material, the reaction was quenched with 20 mL of saturated aqueous $NaHCO_3$, diluted with an additional 20 mL of DCM. The layers were separated and the organic layer was further washed (2x) with 10 mL of of saturated aqueous $NaHCO_3$. The organic layer was then dried *in vacuo* and purified via silica gel column chromatography (100% EtOAc to 5% MeOH:EtOAc) to yield 51 mg (48% yield) of the desired sulfone as a white solid.

^{1}H NMR (400 MHz, $CDCl_3$) δ 8.86 (dd, *J* = 4.8, 1.6 Hz, 1H), 8.26 (dd, *J* = 7.9, 1.6 Hz, 1H), 7.70 (dd, *J* = 7.9, 4.8 Hz, 1H), 3.39 (s, 3H).

^{13}C NMR (101 MHz, $CDCl_3$) δ 159.8, 151.8, 143.8, 126.8, 113.2, 107.4, 40.1.

HRMS High accuracy (ASAP): Calculated for $C_7H_6N_2O_2S$ $(M+H)^+$: 183.0228; found: 183.0223.

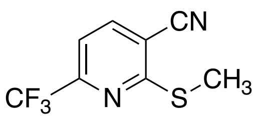

2-(methylthio)-6-(trifluoromethyl)nicotinonitrile (NT-1) The title compound was prepared following the published procedure[10], the product (yellow solid) was carried towards the synthesis of **NT-1-32** without further purification.

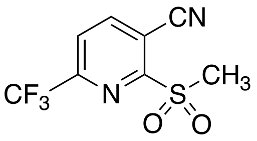

2-(methylsulfonyl)-6-(trifluoromethyl)nicotinonitrile (**NT-1-32**) The title compound was prepared from **NT-1** according to a modified procedure from the literature[11].

Pyridine **NT-1** (105 mg, 0.50 mmol, 1 equiv) was weighed into 50 mL round bottom flask equipped with a stir bar. The solid was dissolved in 10 mL of anhydrous MeOH, followed by portion-wise (usually 3 portions) additions of mCPBA (0.86 mg, 5 mmol, 10 equiv). Substrate conversion was monitored via TLC analysis (40% EtOAc:Hex to 60% EtOAc). Upon complete conversion of starting material, the reaction was quenched with 20 mL of saturated aqueous $NaHCO_3$, diluted with an additional 20 mL of DCM. The layers were separated and the organic layer was further washed (2x) with 10 mL of of saturated aqueous $NaHCO_3$. The organic layer was then dried *in vacuo* and purified via silica gel column chromatography (40% to 80% EtOAc:Hex) to yield 38 mg (30% yield) of the desired sulfone as a white solid.

^{1}H NMR (400 MHz, $CDCl_3$) δ 8.49 (dd, *J* = 8.1, 0.7 Hz, 1H), 8.06 (d, *J* = 8.1 Hz, 1H).

^{13}C NMR (101 MHz, $CDCl_3$) δ 160.4, 149.7 (q, *J* = 37.6 Hz), 146.0, 123.7 (q, *J* = 2.3 Hz), 120.0 (q, *J* = 275.5 Hz), 112.1, 109.7, 39.7.

^{19}F NMR (376 MHz, $CDCl_3$) δ -68.28.

HRMS High accuracy (ASAP)**:** Calculated for $C_8H_5F_3N_2O_2S$ $(M+H)^+$: 251.0102; found: 251.0104.

Preparation of sulfonamide analogs

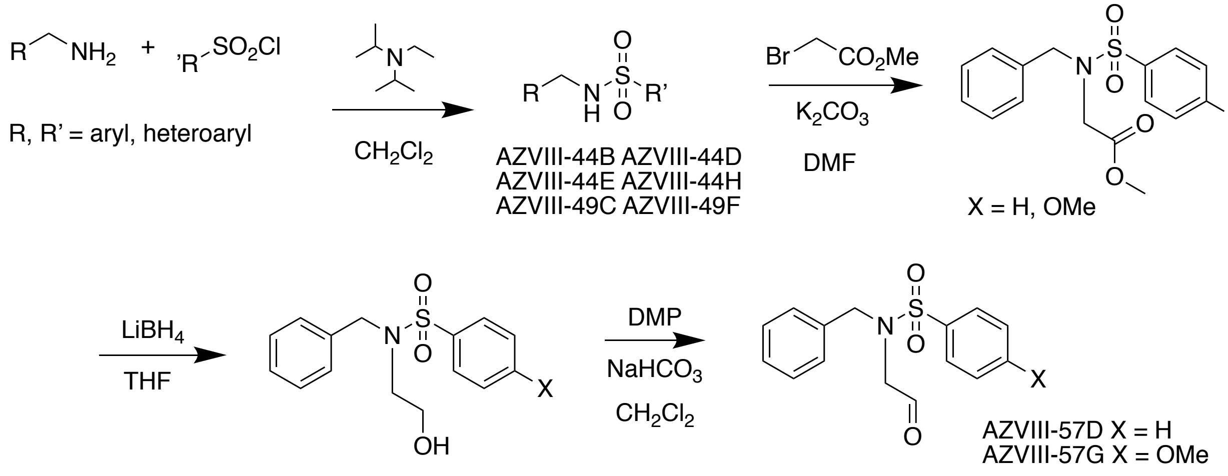

AZVIII-44B, AZVIII-44D, AZVIII-44E, AZVIII-44H, AZVIII-49C, and AZVIII-49F were synthesized by treatment of the appropriate arylalkylamine or heteroarylalkylamine with the corresponding arylsulfonyl chloride or heteroarylsulfonyl chloride and diisopropylethylamine in dichloromethane. AZVIII-57D was synthesized by treatment of AZVIII-44D with methyl bromoacetate and potassium carbonate in dimethylformamide to give the corresponding ester which was reduced to the corresponding alcohol by treatment with lithium borohydride in tetrahydrofuran. The alcohol was then treated with 1,1,1-tris(acetyloxy)-1,1-dihydro-1,2-benziodoxol-3-(1*H*)-one and sodium bicarbonate in dichloromethane to give to the corresponding aldehyde AZVIII-57D. AZVIII-57G was synthesized from *N*-benzyl-4-methoxybenzenesulfonamide according to the procedure used to synthesize AZVIII-57D.

Preparation of compound 4 and its analogs

Compound 4 was synthesized using the synthesis route previously described, with the exception of using a sodium borohydride-cobaltous chloride reduction of the nitrile in the

construction of the lactam, thus avoiding the high pressure hydrogenation in the original route[12]. AZVIII-34D was formed as a byproduct (15%) in the synthesis of compound 4 and was isolated by RP HPLC. ^{1}H NMR (400 MHz, $CDCl_3$) 7.88 (s, 1H), 7.44-7.34 (m, 5H), 6.80 (d, 1H, J = 15.4 Hz), 6.69 (s, 1H), 6.16-5.87 (m, 2H), 5.12 (s, 2H), 4.76 (s, 1H), 4.42 (s, 1H), 4.31-3.99 (m, 4H), 3.39 (s, 2H), 2.40-1.45 (m, 8H), 1.29–1.20 (m, 12H), 1.13-1.02 (m, 3H), 1.00 – 0.89 (m, 6H). MS M+H = 631.

AZVIII-58/GC373: To a solution of 5.31 mg of GC376 in 200 μL H_2O was added 2 μL of saturated $NaHCO_3$. The cloudy mixture was extracted with CH_2Cl_2, dried over Na_2SO_4, and concentrated in vacuum to give the aldehyde (GC373) as a colorless oil (4.0 mg).

AZVIII-38, AZVIII-30, and AZVIII-42 were synthesized according to the procedures described[13]. AZVIII-37A was synthesized as previously described[14]. AZVIII-33B was synthesized according to the method previously described[15].

AZVIII-41A was synthesized according to the procedure described for synthesizing AZVIII-33B[15] and substituting Z-Leu-OH for BOC-Leu-OH. ^{1}H NMR (400 MHz, $CDCl_3$) 7.77 (s, 1H), 7.41-7.27 (m, 5H), 6.80 (dd, 1H, J = 5.5, 15. 6 Hz), 5.98-5.84 (m, 2H), 5.15-5.03 (m, 2H), 4.55 (bd s, 1H), 4.27 (bd s, 1H), 4.23-4.07 (m, 3H), 3.38-3.23 (m, 2H), 2.56–1.40 (m, 8H), 1.33 – 1.20 (m, 3H), 0.95 (s, 6H). MS M+H = 474.

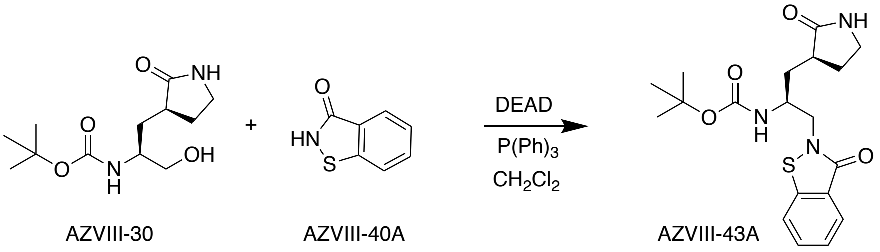

AZVII-43A was synthesized by treatment of a dichloromethane solution of AZVIII-30 and AZVIII-40A with triphenyl phosphine and diethyl azodicarboxylate. ^{1}H NMR (400 MHz, $CDCl_3$) 7.91 (d, 1H, J = 8.0), 7.76 (d, 1H, J = 8.0), 7.55-7.34 (m, 2H), 6.16 (bd s, 1H), 5.29 (bd s, 1H), 4.73-4.39 (m, 2H), 4.35-3.96 (m, 1H), 3.58-3.16 (m, 2H), 3.79-3.43 (m, 2H), 3.39-3.91 (m, 1H), 1.98-1.77 (m, 1H), 1.73-1.57 (m, 1H), 1.42 (s, 9H). MS M+H = 392.

Preparation of GC376 analogs

Glutamine surrogate **5** was synthesized according to previously described procedure and its spectral data were in agreement with the published values[16].

General procedure for synthesis of carbamate **3**

Following the published procedure[17], triphosgene (0.33 equiv.) was added into a cooled (0 °C) vigorously stirred mixture of methyl or ethyl ester of amino acid hydrochloride (2.5 mmol, 1 equiv.) in $NaHCO_3$ (10 mL, sat. aq.sol.) and DCM (10 mL). After stirring at 0 °C for 15 min, the phases were separated and aqueous phase was extracted with DCM (3×). The combined organic layers were dried over $MgSO_4$. Filtration and evaporation of solvents gave isocyanate **1**, which was used in the next step without any further purification.

Following the published procedure[18], isocyanate **1**, alcohol **2** (1 equiv.) and Et_3N (2 equiv.) were refluxed in MeCN (10 mL) for 2 h. The reaction mixture was then concentrated under reduced pressure, diluted by EtOAc, washed with HCl (5%, 1×) and brine (1×), dried over Na_2SO_4, filtered and concentrated under reduced pressure. Column chromatography of the residue on silica gel (hexane/EtOAc) yielded carbamate **3**.

General procedure for synthesis of acid **4**

Following the published procedure[18], carbamate **3** (2 mmol, 1 equiv.) and LiOH (2 equiv.) were stirred in a mixture of H_2O/THF (10 mL, 3/2) for 16 h at rt. Reaction mixture was then concentrated under reduced pressure, diluted by H_2O, acidified by HCl (5%) to pH 2 and extracted with EtOAc (3×). The combined organic layers were then dried over Na_2SO_4. Filtration and evaporation of solvents gave acid **4**, which was used in the next step without any further purification.

General procedure for synthesis of ester **6**

Following the published procedure[18], acid **4** (0.7 mmol, 1 equiv.) and HOBt (1.25 equiv.) were added to the pre-stirred (30 min) solution of EDCI.HCl (1.25 equiv.) and DIPEA (1.4 equiv.) in DMF (8 mL) and the reaction mixture was stirred for 30 min at rt. In a separate flask, a cooled (0°C) solution of glutamine surrogate **5** (1 equiv.) in DMF (2 mL) was treated with DIPEA (4 equiv.). After stirring for 30 min at 0 °C, the glutamine surrogate **5** solution was transferred to the mixture with acid **4** and the resulting reaction mixture was stirred at rt for 15 h. The majority of solvents was evaporated under reduced pressure.

The residue was then diluted by EtOAc, washed with citric acid (10% aq. sol., 3×), $NaHCO_3$ (sat. aq. sol., 1×) and brine (1×), dried over Na_2SO_4, filtered and concentrated under reduced pressure. Column chromatography of the residue on silica gel (DCM/MeOH) yielded ester **6**.

General procedure for synthesis of alcohol **7**

Following the published procedure[18], ester **4** (0.4 mmol, 1 equiv.) and $LiBH_4$ (4 equiv.) were stirred in dry THF (4 mL) and absolute EtOH (1 mL) under Ar atmosphere for 12 h. The reaction was quenched by addition of H_2O and acidified by HCl (5%) to pH 2. The solvent was removed under reduced pressure and the residue was diluted by EtOAc, washed with brine (1×), dried over Na_2SO_4, filtered and evaporated under reduced pressure. Column chromatography of the residue on silica gel (DCM/MeOH) yielded alcohol **7**.

General procedure for synthesis of aldehyde **8**

Following the published procedure[18], DMP (1.5 equiv.) was added to a cooled (0 °C) solution of alcohol **7** (0.3 mmol, 1 equiv.) in dry DCM (5 mL) under Ar atmosphere. The mixture was stirred at 0 °C for 5 min and then at rt for 3 h. Reaction was quenched by addition of a mixture of $Na_2S_2O_3$ (5 mL, 10% aq. sol.) and $NaHCO_3$ (5 mL, sat. aq. sol.). After 5 min stirring at rt, the phases were separated and the aqueous phase was extracted with DCM (3×). The combined organic layers were then washed with brine (2×), dried over Na_2SO_4 and concentrated under reduced pressure. Column chromatography of the residue on silica gel (DCM/MeOH) yielded aldehyde **8**.

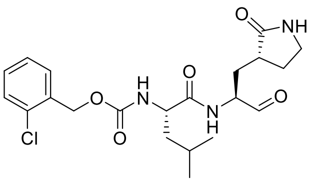

EB33: Yield: 26% in 6 steps. White solid. ^{1}H-NMR (300 MHz, $CDCl_3$) δ 9.49 (s, 1H), 8.34 (d, J = 5.7 Hz, 1H), 7.49–7.31 (m, 2H), 7.29–7.19 (m, 2H overlapping with $CHCl_3$), 6.29 (d, J = 2.6 Hz, 1H), 5.52 (brd, J = 6.8 Hz, 1H), 5.22 (s, 2H), 4.44–4.21 (m, 2H), 3.39–3.20 (m, 2H), 2.53–2.26 (m, 2H), 2.07–1.45 (m, 6H), 1.01–0.86 (m, 6H). ^{13}C-NMR (75 MHz, $CDCl_3$) δ 199.60, 180.07, 173.60, 155.87, 134.14, 133.27, 129.45, 129.37, 129.30, 126.87, 64.13, 57.86, 53.54, 42.31, 40.58, 38.21, 29.67, 28.66, 24.81, 22.92, 21.97.

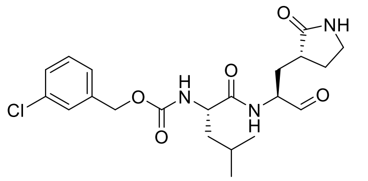

EB34: Yield: 35% in 6 steps. White solid. ^{1}H-NMR (300 MHz, $CDCl_3$) δ 9.48 (s, 1H), 8.44 (d, J = 4.4 Hz, 1H), 7.36 (s, 1H), 7.30–7.25 (m, 2H overlapping with $CHCl_3$), 7.25–7.17 (m, 2H), 5.94 (s, 1H), 5.38 (brd, J = 8.6 Hz, 1H), 5.15–4.99 (m, 2H), 4.41–4.19 (m, 2H), 3.41–3.23 (m, 2H), 2.53–2.28 (m, 2H), 2.01–1.43 (m, 6H), 1.03–0.88 (m, 6H). ^{13}C-NMR (75 MHz, $CDCl_3$) δ 199.77, 180.26, 173.75, 156.00, 138.66, 134.51, 129.93, 128.33, 127.95, 125.97, 66.08 58.14, 53.68, 42.44, 40.76, 38.48, 29.81, 28.89, 24.95, 23.07, 22.07.

General procedure for synthesis of bisulfite adduct **9**

Following the published procedure[18], aldehyde **8** (0.1 mmol, 1 equiv.) and $NaHSO_3$ (1 equiv.) were stirred in a mixture of EtOAc (0.5 mL), EtOH (0.25 mL) and H_2O (0.1 mL) at

50 °C for 3 h. The solvents were then evaporated and the residue was washed with EtOAc (3×). The solid was then dissolved in EtOH, filtered through cotton and dried under reduced pressure. Washing of the solid residue with Et_2O (2×) and mixture of Et_2O/EtOAc (2/1, 1×) yielded bisulfite adduct **9**. The products were obtained as a mixture of two diastereoisomers.

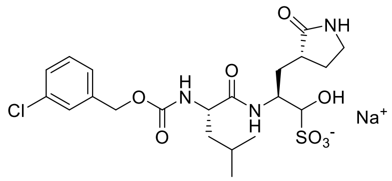

EB46: Yield: 81% from **EB34**. White solid. White solid. ^{1}H-NMR (400 MHz, MeOD) δ 7.41–7.36 (m, 1H), 7.35–7.25 (m, 3H), 5.12 (d, J = 13.0 Hz, 1H), 5.04 (d, J = 13.2 Hz, 1H), 4.45 (d, J = 3.9 Hz, 1H), 4.18–4.09 (m, 1H), 3.99–3.88 (m, 1H), 3.29–3.23 (m, 1H), 3.18 (dd, J = 16.9, 8.6 Hz, 1H), 2.49–2.35 (m, 1H), 2.32–2.19 (m, 1H), 2.04–1.91 (m, 1H), 1.78–1.65 (m, 2H), 1.62–1.49 (m, 3H), 0.96 (d, J = 6.6 Hz, 3H), 0.94 (d, J = 6.6 Hz, 3H). ^{13}C-NMR (125 MHz, MeOD) δ 182.87, 175.71, 158.09, 140.81, 135.33, 131.04, 128.93, 128.47, 126.83, 99.54, 66.52, 66.51, 55.25, 52.83, 52.63, 42.15, 42.12, 41.50, 39.30, 31.26, 30.74, 28.84, 28.80, 25.86, 25.81, 23.33, 23.31, 22.09.

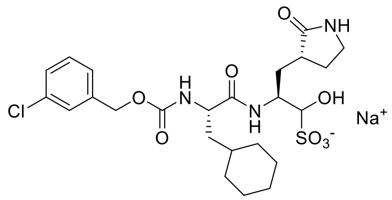

EB48: Yield: 4% in 7 steps. White solid. ^{1}H-NMR (500 MHz, MeOD) δ 7.40–7.37 (m, 1H), 7.35–7.25 (m, 3H), 5.10 (dd, J = 13.1, 2.9 Hz, 1H), 5.06 (dd, J = 13.0, 3.4 Hz, 1H), 4.45 (t, J = 3.9 Hz, 1H), 4.16 (dd, J = 9.5, 5.7 Hz, 1H), 3.99–3.90 (m, 1H), 3.26 (td, J = 13.1,

2.9 Hz, 1H), 3.17 (dd, 13.0, 3.4 Hz, 1H), 2.46–2.36 (m, 1H), 2.31–2.21 (m, 1H), 2.03–1.94 (m, 1H), 1.83–1.47 (m, 9H), 1.34–1.13 (m, 4H), 1.02–0.86 (m, 2H).

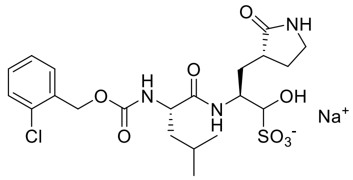

EB54: Yield: 40% from **EB33**. White solid. ^{1}H-NMR (300 MHz, MeOD) δ 7.50–7.44 (m, 1H), 7.42–7.35 (m, 1H), 7.34–7.25 (m, 2H), 5.23 (d, J = 13.4 Hz, 1H), 5.15 (d, J = 13.4 Hz), 4.45 (d, J = 3.8 Hz, 1H), 4.19–4.09 (m, 1H), 4.00–3.86 (m, 1H) 3.25 (td, J = 9.1, 2.2 Hz, 1H), 3.16 (dd, J = 16.8, 8.5 Hz, 1H), 2.49–2.33 (m, 1H), 2.33–2.17 (m, 1H), 2.05–1.19 (m, 1H), 1.80–1.65 (m, 2H), 1.63–1.50 (m, 3H), 0.97 (d, J = 6.9 Hz, 3H), 0.94 (d, J = 7.1 Hz, 3H).

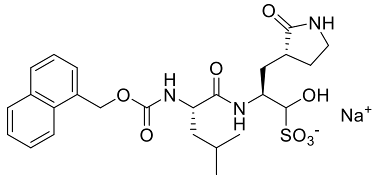

EB55: Yield: 7% in 7 steps. White solid. ^{1}H-NMR (300 MHz, MeOD) δ 8.07 (d, J = 8.1 Hz, 1H), 7.92–7.81 (m, 2H), 7.59–7.40 (m, 4H), 5.64–5.56 (m, 1H), 5.56–5.48 (m, 1H), 4.44 (d, J = 3.4 Hz, 1H), 4.21–4.11 (m, 1H), 4.00–3.85 (m, 1H), 3.28–3.18 (m, 1H), 3.18–3.05 (m, 1H), 2.47–2.32 (m, 1H), 2.32–2.17 (m, 1H), 2.05–1.91 (m, 1H), 1.79–1.46 (m, 1H), 0.93 (d, J = 3.4 Hz, 3H), 0.95 (d, J = 3.4 Hz, 3H).

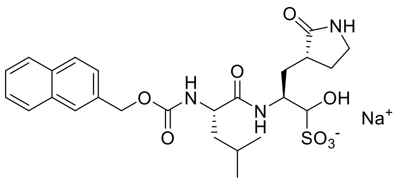

EB56: Yield: 14% in 7 steps. White solid. ^{1}H-NMR (400 MHz, MeOD) δ 7.88–7.81 (m, 4H), 7.52–7.43 (m, 3H), 5.35–5.17 (m, 2H), 4.44 (d, J = 3.6 Hz, 1H), 4.20–4.11 (m, 1H), 4.00–3.87 (m, 1H), 3.18–3.09 (m, 1H), 3.08–2.97 (m, 1H), 2.47–2.33 (m, 1H), 2.25–2.12 (m, 1H), 2.03–1.91 (m, 1H), 1.81–1.45 (m, 5H), 0.96 (d, J = 6.6 Hz, 3H), 0.94 (d, J = 6.5 Hz, 3H).

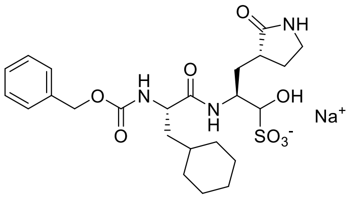

SL-4-241: Yield: 10% in 7 steps. White solid. ^{1}H NMR (400 MHz, MeOH) δ 7.43–7.28 (m, 5H), 5.20–5.07 (m, 2H), 4.55–4.47 (m, 1H), 4.28–4.17 (m, 1H), 4.04–3.94 (m, 1H), 3.36–3.16 (m, 2H), 2.51–2.39 (m, 1H), 2.36–2.27 (m, 1H), 2.09–1.97 (m 1H), 1.88–1.13 (m, 13H), 1.08–0.89 (m, 2H). IR (CD_3OD, cm^{-1}) v 3385, 2924, 2489, 2239, 2073, 1673, 1423, 1345, 1194, 1153, 1117, 971, 901. LCMS (ESI) m/z $[C_{24}H_{34}N_3O_8S]^-$ ($[M]^-$) calculated 524.2, found 524.3.

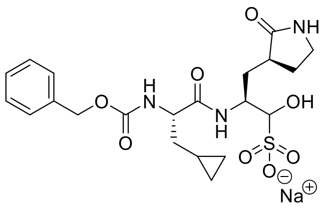

NK01-14: Isolated bisulfite salt as white crystals (0.100 g, 44%) ^{1}H NMR (500 MHz, CD_3OD) δ 7.36 – 7.28 (m, 5H), 5.26 – 5.02 (m, 2H), 4.60 – 4.37 (m, 1H), 4.19 – 4.14 (m, 1H), 3.96 – 3.94 (m, 1H), 3.25 (m, 1H), 3.17 (m, 1H), 2.45 – 2.41 (m, 1H), 2.29 – 2.23 (m,

1H), 2.03 – 1.95 (m, 1H), 1.71 (m, 1H), 1.57 (m, 3H), 0.83 – 0.77 (m, 1H), 0.51 – 0.43 (m, 2H), 0.17 – 0.09 (m, 2H). ^{13}C NMR (126 MHz, CD_3OD) δ 182.85, 175.20, 158.28, 138.26, 129.44, 128.97, 128.74, 99.42, 67.51, 57.31, 52.86, 52.65, 41.48, 39.28, 38.19, 31.22, 30.77, 28.81, 8.56, 5.20, 4.77. HRMS (ESI) $[M-H]^-$: calcd for $C_{21}H_{28}N_3O_8S$: 482.1597; found 482.1630.

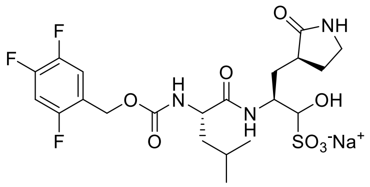

NK01-48: Isolated bisulfite salt as white crystals (0.650 g, 89%) ^{1}H NMR (500 MHz, CD_3OD) δ 7.79 (d, *J* = 7.9 Hz, 1H), 7.58 – 7.27 (m, 1H), 7.22 – 7.16 (m, 1H), 6.97 (t, *J* = 7.3 Hz, 1H), 5.10 (q, *J* = 13.0 Hz, 2H), 4.47 – 4.40 (m, 1H), 4.12 (dd, *J* = 9.2, 6.0 Hz, 1H), 3.98 – 3.92 (m, 1H), 3.33 – 3.25 (m, 1H), 3.23 – 3.18 (m, 1H), 2.54 – 2.37 (m, 1H), 2.32 – 2.24 (m, 1H), 2.03 – 1.93 (m, 1H), 1.79 – 1.66 (m, 2H), 1.56 (ht, *J* = 10.5, 7.6, 5.6 Hz, 3H), 0.96 (d, *J* = 6.5 Hz, 3H), 0.93 (d, *J* = 6.6 Hz, 3H). ^{13}C NMR (126 MHz, CD_3OD) δ 182.86, 175.63, 157.82, 151.14, 148.97, 148.01, 140.28, 129.86, 128.67, 128.36, 122.37, 119.05, 106.60, 99.53, 60.35, 55.28, 52.88, 52.68, 42.13, 41.48, 39.32, 31.30, 30.80, 28.82, 25.84, 23.30, 22.08. ^{19}F NMR (471 MHz, $CDCl_3$) δ -121.35, -136.45, -145.28. HRMS (ESI) $[M+Na]^+$: calcd for $C_{21}H_{27}F_3N_3O_8S$: 480.1722; found 480.1725.

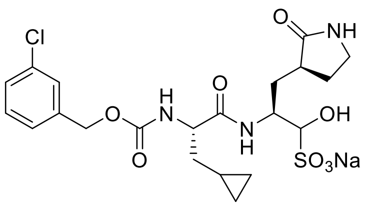

NK01-57: Isolated bisulfite salt as white crystals (0.664 g, 87%) ^{1}H NMR (500 MHz, CD_3OD) δ 7.41 – 7.25 (m, 4H), 5.15 – 5.02 (m, 2H), 4.46 (d, J = 3.7 Hz, 1H), 4.16 (t, J = 7.1 Hz, 1H), 3.96 (ddd, J = 12.7, 8.5, 3.7 Hz, 1H), 3.30 – 3.24 (m, 1H), 3.20 – 3.15 (m, 1H), 2.50 – 2.41 (m, 1H), 2.30 – 2.23 (m, 1H), 1.99 (ddd, J = 15.0, 12.1, 3.5 Hz, 1H), 1.77 – 1.67 (m, 1H), 1.62 – 1.57 (m, 3H), 0.85 – 0.76 (m, 1H), 0.50 – 0.39 (m, 2H), 0.16 – 0.09 (m, 2H). ^{13}C NMR (126 MHz, CD_3OD) δ 182.82, 175.12, 158.03, 157.97, 140.75, 140.26, 135.27, 131.03, 128.92, 128.48, 126.86, 99.42, 66.52, 66.49, 57.34, 57.31, 52.87, 52.66, 41.48, 39.27, 38.20, 38.17, 31.22, 30.80, 28.84, 28.80, 8.55, 5.24, 5.20, 4.80, 4.76. HRMS (ESI) $[M-H]^-$: calcd for $C_{21}H_{27}ClN_3O_8S$: 516.1207; found 516.1207.

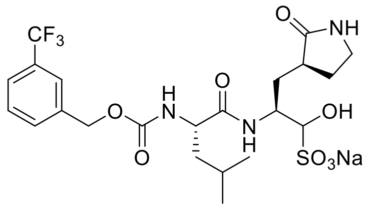

NK01-63: Isolated bisulfite salt as white crystals (0.724 g, 88%) ^{1}H NMR (500 MHz, CD_3OD) δ 7.67 (s, 1H), 7.64 – 7.50 (m, 3H), 5.16 (d, J = 2.6 Hz, 2H), 4.50 – 4.40 (m, 1H), 4.14 (td, J = 7.2, 6.4, 1.6 Hz, 1H), 4.00 – 3.91 (m, 1H), 3.26 (td, J = 9.5, 2.8 Hz, 1H), 3.17 (m, 1H), 2.42 (m, 1H), 2.26 (dddd, J = 15.1, 10.9, 6.4, 2.7 Hz, 1H), 2.12 – 1.94 (m, 1H), 1.71 (m, 2H), 1.65 – 1.50 (m, 3H), 0.94 (dd, J = 14.6, 6.6 Hz, 6H). ^{13}C NMR (126 MHz, CD_3OD) δ 182.79, 175.67, 158.05, 139.83, 132.21, 130.33, 125.57, 125.05, 99.51, 66.53, 64.97, 55.25, 52.83, 52.64, 42.13, 41.46, 39.29, 31.29, 28.79, 25.84, 23.32, 22.05. ^{19}F NMR (471 MHz, $CDCl_3$) δ - 63.13. HRMS (ESI) $[M-H]^-$: calcd for $C_{22}H_{29}F_3N_3O_8S$: 552.1627; found 552.1638.

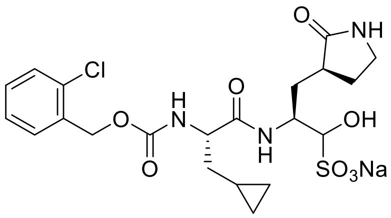

NK01-69: Isolated bisulfite salt as white crystals (0.383 g, 43%) ^{1}H NMR (500 MHz, CD_3OD) δ 7.54 – 7.27 (m, 4H), 5.29 – 5.13 (m, 2H), 4.58 – 4.40 (m, 1H), 4.25 – 4.17 (m, 1H), 4.02 – 3.92 (m, 1H), 3.29 – 3.24 (m, 1H), 3.20 – 3.08 (m, 1H), 2.50 – 2.45 (m, 1H), 2.30 – 2.24 (m, 1H), 2.02 (ddd, J = 15.4, 12.0, 3.5 Hz, 1H), 1.78 – 1.52 (m, 4H), 0.85 – 0.81 (m, 1H), 0.52 – 0.46 (m, 2H), 0.22 – 0.10 (m, 2H). ^{13}C NMR (126 MHz, CD_3OD) δ 182.84, 175.12, 157.95, 135.77, 134.01, 130.48, 130.42, 130.37, 128.15, 99.42, 64.75, 57.38, 52.86, 52.66, 41.47, 39.27, 38.17, 38.15, 28.85, 8.57, 5.21, 4.79. HRMS (ESI) $[M-H]^-$: calcd for $C_{21}H_{27}ClN_3O_8S$: 516.1207; found 516.1213.

Reference of Supplementary Information

1 Iketani, S. *et al.* Lead compounds for the development of SARS-CoV-2 3CL protease inhibitors. *Nat Commun* **12**, 2016, doi:10.1038/s41467-021-22362-2 (2021).

2 Hoffman, R. L. *et al.* Discovery of Ketone-Based Covalent Inhibitors of Coronavirus 3CL Proteases for the Potential Therapeutic Treatment of COVID-19. *J Med Chem* **63**, 12725-12747, doi:10.1021/acs.jmedchem.0c01063 (2020).

3 de Vries, M. *et al.* A Comparative Analysis of SARS-CoV-2 Antivirals Characterizes 3CLpro Inhibitor PF-00835231 as a Potential New Treatment for COVID-19. *Journal of Virology* **95**, e01819-01820, doi:10.1128/JVI.01819-20.

4 Owen Dafydd, R. *et al.* An oral SARS-CoV-2 Mpro inhibitor clinical candidate for the treatment of COVID-19. *Science* **374**, 1586-1593, doi:10.1126/science.abl4784 (2021).

5 Vuong, W. *et al.* Improved SARS-CoV-2 Mpro inhibitors based on feline antiviral drug GC376: Structural enhancements, increased solubility, and micellar studies. *European Journal of Medicinal Chemistry* **222**, 113584, doi:https://doi.org/10.1016/j.ejmech.2021.113584 (2021).

6 Qiao, J. *et al.* SARS-CoV-2 M(pro) inhibitors with antiviral activity in a transgenic mouse model. *Science*, doi:10.1126/science.abf1611 (2021).

7 Su, H. *et al.* Identification of pyrogallol as a warhead in design of covalent inhibitors for the SARS-CoV-2 3CL protease. *Nature Communications* **12**, 3623, doi:10.1038/s41467-021-23751-3 (2021).

8 Vandyck, K. *et al.* ALG-097111, a potent and selective SARS-CoV-2 3-chymotrypsin-like cysteine protease inhibitor exhibits in vivo efficacy in a Syrian Hamster model. *Biochemical and Biophysical Research Communications* **555**, 134-139, doi:https://doi.org/10.1016/j.bbrc.2021.03.096 (2021).

9 Günther, S. *et al.* X-ray screening identifies active site and allosteric inhibitors of SARS-CoV-2 main protease. *Science* **372**, 642, doi:10.1126/science.abf7945 (2021).

10 Metzger, A., Melzig, L., Despotopoulou, C. & Knochel, P. Pd-catalyzed cross-coupling of functionalized organozinc reagents with thiomethyl-substituted heterocycles. *Org Lett* **11**, 4228-4231, doi:10.1021/ol9017003 (2009).

11 Zambaldo, C. *et al.* 2-Sulfonylpyridines as Tunable, Cysteine-Reactive Electrophiles. *J Am Chem Soc* **142**, 8972-8979, doi:10.1021/jacs.0c02721 (2020).

12 Yang, S. *et al.* Synthesis, crystal structure, structure-activity relationships, and antiviral activity of a potent SARS coronavirus 3CL protease inhibitor. *J Med Chem* **49**, 4971-4980, doi:10.1021/jm0603926 (2006).

13 Mou, K. *et al.* Novel CADD-based peptidyl vinyl ester derivatives as potential proteasome inhibitors. *Bioorg Med Chem Lett* **18**, 2198-2202, doi:10.1016/j.bmcl.2007.12.077 (2008).

14 Prior, A. M. *et al.* Design, synthesis, and bioevaluation of viral 3C and 3C-like protease inhibitors. *Bioorg Med Chem Lett* **23**, 6317-6320, doi:10.1016/j.bmcl.2013.09.070 (2013).

15 Amblard, F. *et al.* Synthesis and antiviral evaluation of novel peptidomimetics as norovirus protease inhibitors. *Bioorg Med Chem Lett* **28**, 2165-2170, doi:10.1016/j.bmcl.2018.05.012 (2018).

16 Zhai, Y. *et al.* Cyanohydrin as an Anchoring Group for Potent and Selective Inhibitors of Enterovirus 71 3C Protease. *J Med Chem* **58**, 9414-9420, doi:10.1021/acs.jmedchem.5b01013 (2015).

17 Su, L. *et al.* Design, synthesis and biological evaluation of novel amino acid ureido derivatives as aminopeptidase N/CD13 inhibitors. *Bioorg Med Chem* **20**, 3807-3815, doi:10.1016/j.bmc.2012.04.035 (2012).

18 Galasiti Kankanamalage, A. C. *et al.* Structure-guided design and optimization of dipeptidyl inhibitors of norovirus 3CL protease. Structure-activity relationships and biochemical, X-ray crystallographic, cell-based, and in vivo studies. *J Med Chem* **58**, 3144-3155, doi:10.1021/jm5019934 (2015).
