# Supplementary material for: Development of optimized drug-like small molecule inhibitors of the SARS-CoV-2 3CL protease for treatment of COVID-19
Source: Nat Commun. 2022 Apr 7;13:1891. doi: 10.1038/s41467-022-29413-2 (PMC8989888; doi:10.1038/s41467-022-29413-2)
Supplement: Supplementary file 3 — Reporting Summary [file 41467_2022_29413_MOESM3_ESM.pdf]

nature research

Corresponding author(s): Tomislav Rovis, Alejandro Chavez, David D. Ho, and Brent R. Stockwell

Last updated by author(s): Feb 28, 2022

Reporting Summary

Nature Research wishes to improve the reproducibility of the work that we publish. This form provides structure for consistency and transparency in reporting. For further information on Nature Research policies, see our Editorial Policies and the Editorial Policy Checklist.

Statistics

For all statistical analyses, confirm that the following items are present in the figure legend, table legend, main text, or Methods section.

n/a	Confirmed	
☐	☒	The exact sample size (n) for each experimental group/condition, given as a discrete number and unit of measurement
☐	☒	A statement on whether measurements were taken from distinct samples or whether the same sample was measured repeatedly
☐	☒	The statistical test(s) used AND whether they are one- or two-sided<br>*Only common tests should be described solely by name; describe more complex techniques in the Methods section.*
☒	☐	A description of all covariates tested
☒	☐	A description of any assumptions or corrections, such as tests of normality and adjustment for multiple comparisons
☐	☒	A full description of the statistical parameters including central tendency (e.g. means) or other basic estimates (e.g. regression coefficient) AND variation (e.g. standard deviation) or associated estimates of uncertainty (e.g. confidence intervals)
☐	☒	For null hypothesis testing, the test statistic (e.g. F, t, r) with confidence intervals, effect sizes, degrees of freedom and P value noted<br>*Give P values as exact values whenever suitable.*
☒	☐	For Bayesian analysis, information on the choice of priors and Markov chain Monte Carlo settings
☒	☐	For hierarchical and complex designs, identification of the appropriate level for tests and full reporting of outcomes
☒	☐	Estimates of effect sizes (e.g. Cohen's d, Pearson's r), indicating how they were calculated

Our web collection on statistics for biologists contains articles on many of the points above.

Software and code

Policy information about availability of computer code

Data collection	Tecan iControl (v.1.10); flexControl (v3.4); MassLynx (v4.2); ITCRUN® (v3.8.0).
Data analysis	flexAnalysis (v3.4); XtalView (v4-1); Coot (v0.8.9.2); Phenix (v1.17.1-3660);Maestro (Schrödinger 2019-1);GraphPad Prism (v8.3.1); Microsoft Excel (v16.35); PyMOL (v2.3.4); NanoAnalyze® (v.3.12.0); UNIFI (version 1.8); CellProfiler (v3.0); Plaque (v2.0)

For manuscripts utilizing custom algorithms or software that are central to the research but not yet described in published literature, software must be made available to editors and reviewers. We strongly encourage code deposition in a community repository (e.g. GitHub). See the Nature Research guidelines for submitting code & software for further information.

Data

Policy information about availability of data

All manuscripts must include a data availability statement. This statement should provide the following information, where applicable:

- Accession codes, unique identifiers, or web links for publicly available datasets
- A list of figures that have associated raw data
- A description of any restrictions on data availability

Structural data for the SARS-CoV-2 3CL protease in complex with EB46, EB48, EB54, EB56, SL-4-241, NK01-14, NK01-48, and NK01-63 were deposited in the Protein Data Bank (PDB), with accession codes PDB IDs: 7TIU (https://www.rcsb.org/structure/7TIU), 7TIV (https://www.rcsb.org/structure/7TIV), 7TIW (https://www.rcsb.org/structure/7TIW), 7TIX (https://www.rcsb.org/structure/7TIX), 7TJ0 (https://www.rcsb.org/structure/7TJ0), 7TIA (https://www.rcsb.org/structure/7TIA), 7TIY (https://www.rcsb.org/structure/7TIY), and 7TIZ (https://www.rcsb.org/structure/7TIZ). All other data generated in this study are provided in the Supplementary Information and Source Data file. Publicly available dataset used in this study is the crystal structure of SARS-CoV-2 3CL protease in complex with GC376, with accession code PDB ID: 7JSU (https://www.rcsb.org/structure/7JSU). Source data are provided with this paper.

Field-specific reporting

Please select the one below that is the best fit for your research. If you are not sure, read the appropriate sections before making your selection.

- [x] Life sciences
- [ ] Behavioural & social sciences
- [ ] Ecological, evolutionary & environmental sciences

For a reference copy of the document with all sections, see nature.com/documents/nr-reporting-summary-flat.pdf

Life sciences study design

All studies must disclose on these points even when the disclosure is negative.

Sample size

Sample size calculation were performed for in vivo pharmacokinetic study. Using G*Power 3.1 software (Faul, F., Erdfelder, E., Buchner, A., & Lang, A.-G., 2009), by setting alpha = 5% and power = 90%, the effect size achieved with n=3 is 2.94. This suggested a minimal of 3 mice is required per time point per route of administration. To ensure that there are no sex differences, we will employ 4 mice per condition (2 Male and 2 Female). Sample size calculations were not performed for other experiments. All other experiments were performed with three replicates for each treatment group in order to observe variance unless specifically described in the figure legend. In these cases, these sample sizes were selected because the effect sizes were expected to be large enough to obtain statistical significance from small n values based on preliminary pilot experiments and our prior experiences with similar experiments.

Data exclusions

The only exception is for the ITC analysis of EB46 (Extended Data Fig. 10f), where data point #4 on one set of data deviated from the other points leading to a large error and uncertainty in Kd. Data point #4 was therefore removed before data fitting due to the possibility of an air bubble being released concurrent with the end of the injection. Removal of the data point does not alter any conclusions made in this study.

Replication

To ensure reproducibility of experimental findings, each assay was performed at least two times to confirm the results. We confirm all attempts at replication were successful.
In particular, ITC assays (Supplementary Fig. 4, 10d, and 18) were performed at least in biological duplicates.
SARS-CoV-2 3CLpro IC50 measurements (Fig. 1b; Supplementary Fig. 5b, 10c, 14c,d, 16, and 22c) were carried out with at least two biological replicates for each data point and these data were used to calculate mean values.
SARS-CoV-2 3CLpro kinact/Ki measurements (Table 1; Supplementary Fig. 19) were carried out with biological triplicates for each data point and these data were used to calculate mean values.
Protease selectivity panel IC50 measurements (Supplementary Table 1) were carried out with biological duplicates for each data point.
Single-concentration protease activity inhibition assay (SARS 3CL pro or chymotrypsin, Supplementary Fig. 1, 2, 3c,d, 9, 10b, 14b, and 22d) were carried out with three biological replicates for each data point and these data were used to calculate mean values.
Antiviral activity assays (qRT-PCR, shown in Fig. 2a) were performed in three biological replicates.
Antiviral activity assays (CPE-based, shown in Table 1; Supplementary Fig. 17a,b, and 22e) were carried out with three biological replicates.
Cell-based viral protease inhibition assays and cytotoxicity assays for GC376 analogs (Fig. 5a; Supplementary Fig. 10e and 17c) were carried out with four biological replicates for each data point and these data were used to calculate mean values.
Cell-based SARS-CoV-2 3CL protease inhibition assays for MAC-5576 analogs (Supplementary Fig. 10e) were carried out with biological duplicates for each data point and these data were used to calculate mean values.
Metabolic and solubility assays (Fig. 2b, c; Supplementary Fig. 11, 12) were carried out with at least two biological replicates.
In vivo safety experiments were perform in 10 biological replicates (Figure 6A).
In vivo PK experiments were perform in 8 biological replicates (Figure 6B-C).

Randomization

Mice for in vivo safety and pharmacokinetic experiments were randomly divided into corresponding groups of 10 mice (5 male and 5 females per group for safety) or 4 mice (2 males and 2 females per group for PK). Randomization was not relevant to other experiments, quantifications of which at an ensemble level are not subject to biased interpretation regardless of randomization in sample allocation.

Blinding

For NK01-63 safety experiments, the mouse groups were randomly divided. Then the drug treatment, data collection, and result analysis are blinded. In other words, during the experiment process, the personnels performing the IP or PO injections and weight measurements were not informed which group is treated with drug or water vehicle. It was only revealed after completion of the study. Blinding was not performed for other experiments because the data we analyzed are not subject to biased interpretation.

Reporting for specific materials, systems and methods

We require information from authors about some types of materials, experimental systems and methods used in many studies. Here, indicate whether each material, system or method listed is relevant to your study. If you are not sure if a list item applies to your research, read the appropriate section before selecting a response.

Materials & experimental systems

n/a	Involved in the study
☒	☐ Antibodies
☐	☒ Eukaryotic cell lines
☒	☐ Palaeontology and archaeology
☐	☒ Animals and other organisms
☒	☐ Human research participants
☒	☐ Clinical data
☒	☐ Dual use research of concern

Methods

n/a	Involved in the study
☒	☐ ChIP-seq
☒	☐ Flow cytometry
☒	☐ MRI-based neuroimaging

Eukaryotic cell lines

Policy information about cell lines

Cell line source(s)	1. African green monkey origin, Vero E6 from ATCC; 2. Human embryonic kidney, HEK293T from ATCC; 3. Dog kidney, MDCK from ATCC. 4. Human Pancreas, PANC-1 from ATCC. 5. Human liver, Huh-7 from ATCC. 6. Human colon, Caco-2 from ATCC.
Authentication	All cells were from ATCC with authentication. The authentication was performed by morphology check under microscopes and growth curve analysis.
Mycoplasma contamination	We confirm that all cells were tested as mycoplasma negative.
Commonly misidentified lines (See ICLAC register)	No commonly misidentified cell lines were used.

Animals and other organisms

Policy information about studies involving animals; ARRIVE guidelines recommended for reporting animal research

Laboratory animals	This study involves 88 C57BL/6 mice ordered from Jackson Lab. 44 mice are male, while the other 44 are female. Mice were housed 4 per cage under 12h light/dark cycle conditions. Ambient temperature is 22˚C and humidity is 50%. Food and water were provided ad libitum.
Wild animals	This study did not involve wild animals.
Field-collected samples	This study did not involve field-collected samples.
Ethics oversight	Animal study protocol (AC-AAAZ4461) was approved by Columbia IACUC (institutional animal care and use committee) to investigate the safety and PK properties of NK01-63.

Note that full information on the approval of the study protocol must also be provided in the manuscript.
